# Influences of sleep and lifestyle factors on the risk for covid-19 infections, from internet survey of 10,000 Japanese business workers

**DOI:** 10.1038/s41598-022-22105-3

**Published:** 2022-11-16

**Authors:** Masahiro Nakashima, Ryota Amano, Naoya Nishino, Yasutaka Osada, Yuriko Watanabe, Akifumi Miyake, Shintaro Chiba, Seiji Nishino

**Affiliations:** 1 Institute for Research, Brain Sleep, Tokyo, Japan; 2 Research Institute, 3S Capital, Tokyo, Japan; 3 Graduate School of Information Sciences, Tohoku University, Aoba-ku, Sendai, Japan; 4 Department of Psychiatry, Sleep and Circadian Neurobiology Laboratory, Stanford University School of Medicine, Palo Alto, CA USA; 5 Department of Otorhinolaryngology, Jikei University School of Medicine, Tokyo, Japan; 6 Ota Memorial Sleep Center, Ota General Hospital, Kawasaki, Kanagawa Japan; 7 Sleep and Circadian Neurobiology Laboratory, Department of Psychiatry and Behavioral Sciences, Stanford University School of Medicine, 3155 Porter Drive, Rm2106, Palo Alto, CA 94304 USA

**Keywords:** Viral infection, Sleep disorders

## Abstract

We conducted an internet survey to assess sociodemographic variables, lifestyle factors, sleep problems, and comorbidities for sleep apnea syndrome (SAS) in COVID-19 and influenza (FLU) infections. Data from 10,323 workers (50.0% male) were analyzed. COVID-19 was diagnosed in 144 subjects (COVID-19+), and 8,693 were classified as not suspected to be infected (COVID-19−). SAS had been diagnosed in 35.4% of the COVID-19+ subjects, but only 231 (2.7%) of the 8,693 COVID-19− subjects. COVID-19+ subjects were more susceptible to FLU (35.4%) compared to COVID-19− subjects (3.0%). A multivariate analysis revealed that higher risks of COVID-19+ were linked to the following factors: going out without a face mask (OR 7.05, 95% CI 4.53–11.00), FLU+ (OR 6.33, 95% CI 3.80–10.54), excessive exercise before going to sleep (OR 2.10, 95% CI 1.63–2.70), SAS+ (OR 5.08, 95% CI 2.88–8.94), younger age (OR 1.05, 95% CI 1.03–1.07), falling sleep while sitting or talking with someone (OR 3.70, 95% CI 2.30–5.95), and use of hypnotics (OR 2.28, 95% CI 1.20–4.30). Since sleep impairment played a relatively small role in COVID-19+/SAS− subjects, we assume that SAS itself was a more significant risk factor for COVID-19 infection rather than sleep impairment. A better understanding of the mechanisms that result in increased susceptibility to COVID-19 in SAS is vital for helping prevent COVID-19.

## Introduction

The importance of sleep problems on health and disease has been emphasized in recent decades^1,2^. In addition to well-known functions of sleep, such as (a) restoration of sleepiness and fatigue^3^, (b) memory fixation^4^, (c) hormone and autonomic nerve adjustments^5^, sleep has also recently been recognized for (d) strengthening immune functions^6,7^ and for (e) facilitating the clearance of waste products in the brain^8^.

Although public awareness of the importance of sleep has increased recently, people living in the modern era, typically represented by business workers, tend to stay up late and sleep less, resulting in chronic sleep loss^9^. Chronic sleep loss impairs not only work performance, but also increases the risk for various diseases, including metabolic syndrome, infections, hypertension, strokes, ischemic heart diseases, psychiatric diseases, cancers, and cognitive disorders^10^.

COVID-19 incidences have emphasized the importance of sleep since poor sleep negatively affects natural immunity and increases the risk of acquiring infections, including common colds and seasonal influenza^6^. Inadequate sleep also impairs acquired immunity and reduces the effectiveness of vaccinations, as well as delays recovery from illnesses^7^. COVID-19 lockdowns have also been linked to changes in sleep schedules, quantity and quality of night-time sleep, which may have an additional impact on infection risks^11^. Additionally, recent studies suggest that patients with obstructive sleep apnea (OSA), a sleep disorder characterized by frequent respiratory arrests and disturbed sleep, have a significantly increased risk of developing COVID-19 infection, as well as hospitalization and mortality caused by COVID-19^12,13^.

OSA has been hypothesized to increase COVID-19 severity through proinflammatory pathways which occur as a consequence of the disease^14,15^. According to the study by Marrs et al., OSA poses one of the greatest risks for COVID-19 infections, where the prevalence of COVID-19 infection is eight times higher than in the general population^16^.

However, the majority of previous studies on the association between diagnosed OSA and COVID-19 have so far examined COVID-19-hospitalized patients with retrospectively examined medical records of OSA^12,13,16^, and only a few studies on OSA and COVID-19 in the general population, such as by Chung et al., are available^17^.

We are particularly interested in the research of office-based workers, because many of them have chronic sleep loss or problems and have experienced work habit changes, such as remote work, during the COVID-19 pandemic. Therefore, in the current study we examined the association between sleep problems and other lifestyle factors, comorbidities for sleep and other diseases, and the COVID-19 infection risk of 10,000 Japanese office workers.

## Results

**Figure 1:**
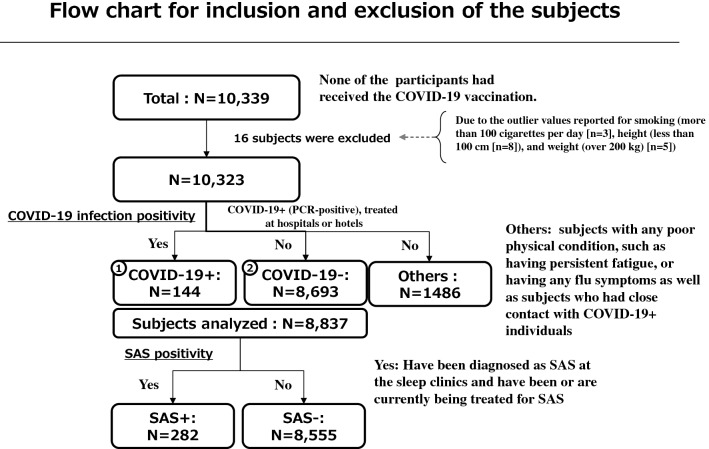
Flow chart for inclusion and exclusion of the subjects.

10,339 participants were initially enrolled in the survey (Fig. 1). Data for 16 subjects were excluded due to outlier values reported for smoking (more than 100 cigarettes per day [n = 3]), height (less than 100 cm [n = 8]), and weight (over 200 kg [n = 5]), possibly due to input errors. The data from 10,323 subjects (42.0 ± 13.0 years, 50.0% male) was then analyzed. 144 (32.6 ± 11.8 years, 74.3% male) were identified to be COVID-19+ (Tables 1, S1). Of the remaining 10,179 subjects, 1486 had an uncertain infection status, thereby being excluded from COVID-19− group (8693, 42.8 ± 12.9 years, 48.9% male), resulting in an analysis of 8837 total subjects (42.6 ± 13.0 years, 49.3% male) for the comparison (Fig. 1, Table 1).

**Table 1:** Component ratio for the participants for the analysis (n = 8837).

Age	COVID-19+	COVID-19−	COVID19+/COVID-19− (%)	SAS+	SAS−	SAS+/SAS−19− (%)
**Male**						
20 ~	76	1051	7.2	60	1067	5.6
30 ~	6	529	1.1	14	521	2.7
40 ~	11	1055	1.0	57	1009	5.6
50 ~	10	1046	1.0	66	990	6.7
60 ~	4	567	0.7	41	530	7.7
All	(103)	(3681)	(2.8)	(197)	(3587)	(5.5)
**Female**						
20 ~	21	1213	1.7	15	1219	1.2
30 ~	6	813	0.7	6	813	0.7
40 ~	4	799	0.5	5	798	0.6
50 ~	4	1318	0.3	13	1309	1.0
60 ~	2	302	0.7	5	299	1.7
All	(35)	(4143)	(0.8)	(39)	(4143)	(0.9)
Total	144	8693	1.6	282	8555	3.2

We found that a large majority of COVID-19+ patients were in their 20 s, and that males were more susceptible than females, which is consistent with the epidemiological data for COVID-19 in Japan^18^. The medical history of sleep apnea syndrome (SAS) was specifically asked if “you have ever been diagnosed with and treated for SAS at a hospital or medical institution” (see, Supplement: Survey questionnaires Q15). In terms of SAS+ distribution, males were more susceptible, with a tendency for age groups in the 20 s and over 60 to be more affected in both sexes (Table 1). There were no significant differences in BMI between the COVID-19+ and COVID-19− groups overall (Table 2). Significantly higher BMI was observed in SAS+ (compared to SAS−) in both males and females over 40, but this was not apparent in SAS+ subjects in their 20 s and 30 s (Table 2).

**Table 2:** BMI (mean ± SD) values and COVID/Sleep Apnea status in each age group.

Age	COVID-19+	COVID-19−	COVID19+/COVID-19− (%)	SAS+	SAS−	SAS+/SAS− (%)
**Male**						
20 ~	22.4 ± 4.5	22.2 ± 3.7	1.01	23.0 ± 5.3	22.2 ± 3.6	1.04
30 ~	23.7 ± 3.2	23.0 ± 4.1	1.03	23.9 ± 9.9	23.0 ± 3.8	1.04
40 ~	23.0 ± 2.8	23.6 ± 3.6	0.97	26.2 ± 4.6**	23.5 ± 3.4	1.11
50 ~	24.9 ± 4.1	24.0 ± 3.7	1.04	26.3 ± 3.8**	23.8 ± 3.6	1.11
60 ~	21.8 ± 2.4	23.7 ± 3.3	0.92	24.5 ± 4.6*	23.6 ± 3.2	1.04
All	22.7 ± 4.2	23.3 ± 3.7	0.98	25.0 ± 5.2**	23.2 ± 3.6	1.08
**Female**						
20 ~	19.0 ± 4.7	20.5 ± 3.2	0.93	21.6 ± 3.2	20.5 ± 3.3	1.06
30 ~	21.9 ± 4.4	21.1 ± 3.7	1.04	22.1 ± 4.0	21.1 ± 3.9	1.05
40 ~	18.3 ± 1.6*	21.7 ± 3.6	0.85	23.5 ± 4.6	21.7 ± 3.7	1.09
50 ~	21.5 ± 2.3	21.7 ± 3.6	0.99	25.8 ± 4.1**	21.7 ± 3.6	1.19
60 ~	25.6 ± 2.0	21.7 ± 3.2	1.18	22.0 ± 4.3*	21.7 ± 3.2	1.01
All	20.0 ± 4.4	21.3 ± 3.6	0.94	23.2 ± 4.1**	21.2 ± 3.6	1.09
Total	22.0 ± 4.4	22.0 ± 4.4	0.99	24.7 ± 5.1**	22.2 ± 3.7	1.12

The values are displayed mean ± SD.

**p < 0.01, * p < 0.05, Mann–Whitney U test.

51 (35.4%) of 144 COVID-19+ subjects claimed to have been diagnosed and treated for SAS, while only 231 (2.7%) of 8693 COVID-19− subjects confirmed that they had been diagnosed and treated (χ2 = 481.5, p < 0.001) (Table 3). COVID-19+ subjects were also more susceptible to FLU; 51 (35.4%) of 144 COVID-19+ subjects were infected with FLU, compared to only 264 (3.0%) of 8693 COVID-19− subjects (χ^2^ = 422.7, p < 0.001) (Table 4). Hence SAS+ were more susceptible to FLU infection (13.3%) than SAS− subjects (2.8%) (χ^2^ = 105.4, p < 0.001) (Table5).

**Table 3:** Cross tabulation table for COVID-19 and SAS positivity (3a: all age, 3b: 20 s).

	3a				3b			
	COVID-19+		COVID-19−		COVID-19+		COVID-19−	
	Number	%	Number	%	Number	%	Number	%
SAS+	51	35.4	231	2.7	41	42.3	34	1.5
SAS−	93	64.6	8462	97.3	56	57.7	2230	98.5
	X^2^ = 481.5, P < 0.001				X^2^ = 489.4, P < 0.001

**Table 4:** Cross tabulation table for COVID-19 and FLU positivity (4a: all age, 4b: 20 s).

	4a				4b			
	COVID-19+		COVID-19−		COVID-19+		COVID-19−	
	Number	%	Number	%	Number	%	Number	%
FLU+	51	35.4	264	3.0	38	39.2	78	3.4
FLU−	93	64.6	8429	97.0	59	60.8	2186	96.6
	X^2^ = 422.7, P < 0.001				X^2^ = 246.6, P < 0.001

**Table 5:** Cross tabulation table for FLU and SAS positivity (5a: all age, 5b: 20 s).

	5a				5b			
	FLU+		FLU−		FLU+		FLU−	
	Number	%	Number	%	Number	%	number	%
SAS+	42	13.3	240	2.8	28	24.1	47	2.1
SAS−	273	86.7	8282	97.2	88	75.9	2198	97.9
	X^2^ = 105.4, P < 0.001				X^2^ = 167.2, P < 0.001			

Since the majority of COVID-19+ were in their 20 s and the mean age of COVID-19+ (32.6 ± 11.8 years) was significantly lower than that of COVID-19− (42.6 ± 13.0 years, p < 0.001), we also repeated the same cross tabulation analysis with only subjects in their 20 s. We found similar results in this selected population (Tables 3, 4 and 5).

Univariate analysis revealed that age, sex, BMI, and some sociodemographic factors (job, income), work performance levels and lifestyle habits (smoking, bathing, drinking before going to bed, exercising excessively before going to bed), method of avoiding COVID-19 infection (wearing a face mask outdoors), and sleep parameters (falling sleep while sitting or talking with someone, oversleeping) were significant for COVID-19 or SAS positivity in all ages and 20 s (see, Supplement S4-1-S4-8).

Multivariable logistic regression analyses were then applied with 8 to 12 variables, depending on statistical point of view and clinical importance, as well as the number of subjects used in each analysis to examine the association between the risk of COVID-19+, SAS+ (Tables 6, 7, S3-1, and S3-2). We confirmed that the sample size of the lesser category of dependent variables should be 10 times or larger to avoid bias, precision, and significant testing issues in logistic regression^19^. The VIF (Variance Inflation Factor), a measure of multicollinearity, has been confirmed to be less than 2 for all variables. As a result, the negative impact of multicollinearity is thought to be extremely small.

**Table 6:** Odd ratio and 95% confidence interval for the significant association of the variables with COVID-19+, all age.

Variables	Coeff	z value	OR	95% confidence interval		p value
				Lower	Upper	
Going out without face mask	1.95	8.63	7.05	4.53	11.00	**6.22E−18**
FLU+	1.84	7.09	6.33	3.80	10.54	**1.39E−12**
Excessive exercise before going to bed	0.74	0.48	2.10	1.63	2.70	**1.14E−08**
SAS+	1.62	5.63	5.08	2.88	8.94	**1.79E−08**
AGE (younger)	0.05	0.95	1.05	1.03	1.07	**4.46E−08**
Falling asleep while sitting or talking with someone	1.31	0.27	3.70	2.30	5.95	**6.69E−08**
Use of hypnotics	0.82	2.53	2.28	1.20	4.30	**0.011**
SEX (male)	0.45	0.64	1.56	0.98	2.49	0.059
Severe sleepiness while driving, eating meals, engaging in social activity	0.45	1.58	1.57	0.90	2.75	0.11
BMI	0.02	0.98	1.02	0.98	1.07	0.33
Oversleep (Getting up late)	0.09	0.91	1.10	0.86	1.41	0.45
Sleep longer during weekend	0.03	0.97	1.03	0.66	1.61	0.90

Significant values are in bold.

**Table 7:** Odd ratio and 95% confidence interval for the significant association of the variables with SAS+, all age.

Variables	Coeff	z value	OR	95% confidence interval		p value
				Lower	Upper	
COVID-19+	2.48	10.28	11.95	7.45	19.17	**8.47E−25**
SEX (male)	1.39	8.03	4.00	2.85	5.62	**9.62E−16**
BMI	0.10	7.07	1.11	1.08	1.14	**1.58E−12**
Work performance (impaired)	0.00	5.91	1.00	1.00	1.01	**3.50E−09**
AGE (older)	0.33	5.01	1.39	1.22	1.59	**5.38E−07**
Sleep right after eating a meal	0.03	4.90	1.03	1.02	1.04	**9.56E−07**
Remote work (working from home)	0.17	4.40	1.19	1.10	1.29	**1.08E−05**
Acknowledging oneself as a short sleeper	0.59	4.40	1.81	1.39	2.36	**1.10E−05**
Sleep in a room with the TV/light on	0.11	1.85	1.12	0.99	1.26	0.064

Significant values are in bold.

The multivariate logistic analysis for 8837 subjects (all age groups) revealed that higher risks for COVID-19+ were associated with going out without a face mask (OR 7.05, 95% CI 4.53–11.00), FLU+ (OR 6.33, 95% CI 3.80–10.54), excessive exercise before going to bed (OR 2.10, 95% CI 1.63–2.70), SAS+ (OR 5.08, 95% CI 2.88–8.94), younger age (OR 1.05, 95% CI 1.03–1.07), falling asleep while sitting and talking to people (OR 3.70, 95% CI 2.30–5.95), and use of hypnotics (OR 2.28, 95% CI 1.20–4.30) (Table 6).

High risks for SAS+ were associated with COVID-19+ (OR 11.95, 95% CI 7.45–19.17), male gender (OR 4.00, 95% CI 2.85–5.62), higher BMI (OR 1.11, 95% CI 1.08–1.14), impaired work performance (OR 1.00, 95% CI 1.00–1.01), older age (OR 1.39, 95% CI 1.22–1.14), sleeping right after eating a meal (OR 1.39, 95% CI 1.22–1.59), remote work (OR 1.19, 95% CI 1.10–1.29), and self-reported as a short sleeper (OR 1.81, 95% CI 1.39–2.36) (Table 7).

Multivariate logistic analysis for 2361 of the 20 s age group revealed that higher risks for COVID-19+ were associated with going out without a face mask (OR 11.57, 95% CI 6.30–21.20), FLU+ ( OR 6.48, 95% CI 3.18–13.20), SAS+ (OR 7.34, 95% CI 3.44–15.68), excessive exercise before going to bed (OR 2.13, 95% CI 1.52–3.03), tendency for dozing off (OR 3.64, 95% CI 1.94–6.83), use of hypnotics (OR 3.25, 95% CI 1.27–8.32) and male gender (OR 1.92, 95% CI 1.01–3.65) (Supplemental S3-1).

High risks for SAS+ in the 20 s age group were associated with COVID-19+ (OR 10.90, 95% CI 5.33–22.31), self-reported as a short sleeper (OR 6.15, 95% CI 3.30–11.44), impaired work performance (OR 1.01, 95% CI 1.00–1.01), remote work (OR 1.32, 95% CI 1.09–1.60), sleeping in a room with TV/light on (OR 1.61, 95% CI 1.16–2.23), sleeping right after eating a meal (OR 1.45, 95% CI 1.10–1.92), and higher BMI (OR 10.7, 95% CI 1.00–1.14) (Supplemental S3-2).

## Discussion

Subjects with medical history of SAS have a significantly increased risk for COVID-19 infection; 51 (35.4%) of 144 COVID-19+ subjects had been diagnosed and treated with SAS, compared to only 231 (2.7%) of 8693 COVID-19− subjects (i.e., 13-fold greater risk) (Table 3). While the difference was surprisingly large, our findings are consistent with those from a report by Maas et al.^16^ where the authors reported that patients with OSA in the United States experienced an approximately eightfold higher risk for COVID-19 infection compared to a similar age group receiving care in a large, racially and socioeconomically diverse healthcare system. Since the prevalence of COVID-19 was evaluated in the designated OSA subjects and in the data of the health care system respectively, several authors raised questions about the accuracy of the data comparison^20^. They especially questioned the low prevalence of OSA (0.8%) in the control group (vs. 6.3% in the OSA patient’s group), while the prevalence of OSA in the United States has been reported to be 3–7%^21^.

The prevalence of SAS was 3.2% (282/8837) in our study, which is well within the range of SAS prevalence in Japan^22^. With regards to the COVID-19+ status, none of the participants had received the COVID-19 vaccine during the research period, and a positive polymerase chain reaction result for SARS-CoV-2 was confirmed prior to hospitalization and hotel therapy as per government standards^18^. In contrast, COVID-19− statuses were self-reported, and it is possible that some subjects with subclinical infection were included in our COVID-19– group. Therefore, we attempted to exclude subjects with any poor physical condition, such as persistent fatigue or flu symptoms (sore throat, cough, sputum, and fever) from January 2020 to the time subjects answered the questionnaire, from the COVID-19− group. In addition, subjects who had visited clinics and undergone home isolation due to COVID-19+ close contact, as well as subjects whose relatives, friends, or coworkers had been diagnosed with COVID-19+ were excluded from the COVID-19− group. Although contamination of subclinical COVID-19+ cases may still occur in the COVID-19− group, we believe this would increase the SAS prevalence in the COVID-19− group rather than overestimate the odds ratio for SAS positivity for the risk for COVID-19+.

A recent cross-sectional population-based web survey of 20,598 adults from 14 countries/regions found that having a high risk for OSA (evaluated with a standardized questionnaire and 9.5% prevalence) was linked to an increased risk of COVID-19 hospitalization or ICU treatment (OR 2.11, 95% CI 1.10–4.01)^17^. Furthermore, male gender (OR 2.82, 95% CI 1.55–5.12), diabetes (OR: 3.93, 95% CI 1.70–9.12), and depression (OR: 2.33, 95% CI 1.15–4.77) were found to be associated with an increased risk of COVID-19 hospitalization or ICU treatment^17^. Several other non-population-based studies have also reported an increased risk of COVID-19 infections in OSA patients, with consistent results, though the risk-odds ratio reported varied depending on the study^12,13,16^.

As previously reported^18^, a large majority of COVID-19+ subjects were in their 20 s (67.4%) and a relatively large number of subjects with a medical history of SAS were in their 20 s as well (26.6%). Nishijima et al.^23^, recently reported that the prevalence of OSA in young adults in Japan, particularly males under 30 years old, is comparable to or even higher than that in older age groups (23). The negative impacts of SAS and other sleep disorders on health and disease have become more widely recognized in Japan in recent years. Consequently, young people frequently visit sleep clinics, and more SAS cases are likely to be diagnosed as a result. Furthermore, some public or commercial transportation companies require an SAS examination as part of the job application process (Dr. Chiba, a personal communication). These circumstances may explain why there are so many young SAS subjects. Nonetheless, in our study we also found that older age is still one of the risk factors for SAS (Table 7). However, because a large majority of COVID-19+ subjects were in their 20 s and the mean age of COVID-19+ was significantly younger than that of COVID-19−, we also analyzed the data set of only 20 s to avoid potential confounding factors related to aging. We discovered very similar results for this selected population, which corresponded to the findings for all ages (Tables 3, 4, 5, 6,7, S3-1, and S3-2).

We found that COVID-19+ subjects were also more susceptible to FLU. 35.4% of 144 COVID-19+ subjects were infected with FLU, while only 3.0% of 8693 COVID-19− subjects were infected (χ^2^ = 422.7, p < 0.001) (Table 4). Because COVID-19+ and FLU+ were not exclusive to each other, the vulnerability to upper airway viral infections was most likely involved in both infections, with SAS being the risk factor for both infections (Tables, 3 and 5).

The multivariate logistic analysis for 8837 subjects revealed that high risk for COVID-19+ was associated with going out without a face mask (OR 7.05), FLU+ (OR 6.33), excessive exercise before going to bed (OR 2.10), SAS+ (OR 5.08), younger age (OR 1.05), dozing off [falling asleep while sitting and talking with someone] (OR 3.70), and use of hypnotics (OR 2.28) (Table 6).

We initially hypothesized that impaired sleep decreases the immune function, and that it may result in an increased risk for COVID-19 and FLU infections. This may still be partially true, but having SAS itself appeared to have a much larger influence on COVID-19 infection risk. The sleep indexes (i.e., PSQI) of COVID-19+ (7.2 ± 3.2) were significantly worse than those of COVID-19− (5.4 ± 2.6) (p < 0.001). However, since having SAS contributed significantly to the impaired sleep index of the entire COVID-19+ group (COVID-19+/SAS+: 8.9 ± 2.6, p < 0.001, compared to COVID-19−, Kruskal–Wallis test with Bonferroni test), contributions of sleep impairments for COVID-19+/SAS− (6.3 ± 3.1) are not remarkably significant (p < 0.02, compared to COVID-19−). As a result, the global PSQI sleep index became a non-significant explanatory variable with the multivariate analysis for COVID-19+. Dozing off [falling asleep while sitting and talking with someone] and use of hypnotics remained significant for COVID-19+ in the multivariate analysis. The use of hypnotics could indicate that severe insomnia is a risk factor for COVID-19+. Although it is unclear how excessive exercise before going to bed increases the risk for COVID-19+, a recent study suggests that excessive exercise may reduce immune function^24^. These individuals are more likely to go out at night, which may also affect their sleep and biological rhythms.

The mechanisms underlying the increased risk of COVID-19+ in SAS patients remain largely unknown. As discussed by several authors, angiotensin converting enzyme 2 (ACE2)-mediated mechanisms may be involved, since higher ACE2 activity in SAS has been reported^25^ and both SARS-CoV-2 and influenza viruses infect through the ACE2 protein^26,27^. Although the treatment status of SAS at the time of the survey was not recorded in our study, mouth breathing due to SAS may also increase the risk for upper airway infections, as normal nose breathing adds humidity and warmth to the airflow and also increases nitric oxide levels in the airways, which may decrease viral load and enhance antiviral response during sleep^28^. Proton pump inhibitor (PPI) use has recently been linked to an increased risk of COVID-19+^29^. Because SAS+ patients frequently have gastroesophageal reflux disease (GERD), and GERD is commonly treated with PPIs^30^, it is possible that a PPI-mediated mechanism is also involved. Interestingly, our study also revealed that sleeping immediately after eating a meal is a risk factor for SAS+. Further studies on the mechanisms that increase the risk of COVID-19 infection in SAS patients will be useful in preventing COVID-19 infection for SAS patients and the general public.

Limitations of the study must be addressed. In order to participate in the survey, participants needed access to the internet and the ability to input their answers online, and these factors potentially limit the generalizability of our findings. However, since we focused on office workers and the main results came from the younger population, we believe this does not significantly bias the results. The medical history of SAS in the current study was self-reported. We however specifically asked if “you have ever been diagnosed with and treated for SAS at a hospital or medical institution”. The prevalence of SAS in the current study was 3.2%, which is well within the range of SAS prevalence in Japan^22^, and thus we believe that SAS subjects are not overestimated. We were unable to include subjects who died as a result of severe COVID-19 infection, but because the ratio for these cases is estimated to be very low^18^, the overall outcome of the study would remain unchanged. Due to the limitations of the internet survey, we were also unable to analyze the relationship between severity of SAS, treatment status, and adherence for the COVID-19+ risk. In addition, we did not examine and compare the COVID-19+ risk among SAS, diabetes, hypertension, and depression, all of which have been linked to an increased risk of COVID-19 infection. Nonetheless, our study is the first population-based study to report on SAS and the increased risk of COVID-19 infection in Japanese business workers, and we believe the findings will have substantial value in COVID-19 and SAS epidemiology.

In conclusion, through an internet-based survey of 10,000 Japanese business workers, we identified SAS+, excessive daytime somnolence, use of hypnotics, and FLU+ as risk factors for COVID-19 infection, in addition to the well-known risk factors, such as going out without a mask, and younger age. Given the small contributions for sleep impairment in COVID-19+/SAS− subjects, we believe that SAS itself is a more significant risk factor for COVID-19 infection. The mechanisms for increased susceptibility to COVID-19 and FLU infections in SAS patients are vital to study in order to prevent and better manage COVID-19 infections in the general population.

## Methods

### Survey of sleep and lifestyle parameters, comorbidities during the COVID-19 pandemic

The initial goal for the study was to recruit 10,000 business workers (over the age of 20) for an internet cross-sectional survey for sleep and lifestyle variables, as well as comorbidities during the COVID-19 pandemic. The recruitment and survey were carried out in February 2021, approximately one year after the COVID-19 pandemic had significantly impacted Japan and before COVID-19 vaccinations began in the country.

The internet survey was conducted with the cooperation of one of the largest online survey companies (the "Survey Firm") in Japan. The Survey Firm has approximately 10 million members in Japan and conducts more than 20,000 surveys per year. The Survey Firm collects members from a wide range of media, including affiliate advertising, mailing lists, and literary magazines. Members are pre-registered in the Survey Firm's database with basic demographic information such as location, gender, and annual income. Members are asked to respond to each survey, and are incentivized to do so by earning points that can be redeemed for cash, merchandise, or other rewards.

In the current study, we asked the Survey Firm to collect responses from approximately 10,000 Japanese office workers. The Survey Firm collected responses from members so that the number of respondents in each category for each prefecture, age, and gender would be a pre-determined number, taking into account the demographic composition of the population. The final total of 10,339 responses was collected in the order of the earliest to latest responses.

Participants were drawn from each of Japan’s 47 prefectures (Tables S1-1–S1-4). In accordance with the population distribution ratio, larger numbers of participants (270 to 651 people) were assigned to the 9 populated prefectures, while an equal number of participants (186 people) were assigned to the other 38 prefectures. The study protocol was approved by the ethical committee of Ota General Hospital in Kawasaki, Kanagawa, Japan, and all participants provided informed consent. All procedures were carried out in accordance with the applicable guidelines and regulations.

### Survey parameters

The questionnaires contained 147 items divided into 22 categories, including age, gender, height, weight, body mass index (BMI), sociodemographic questions, such as job and profession, presence of remote work, work performance levels, and lifestyle habits such as activities before going to bed, computer and tablet usage, exercise, drinking, smoking, and bathing, in addition to the Pittsburgh Sleep Quality Index (PSQI) (Q6-12, see, Supplemental Material Tables S1, S2, S4 and S5) and + Epworth Sleepiness Scale (Q18) (see^31^). Medical history of SAS was specifically asked by “whether the subjects have been diagnosed as SAS+ at the sleep clinics and have been or are currently being treated for SAS” (Q13).

Health status, including various indefinite complaints, as well as COVID-19 status (Q27) and prevention approaches used for COVID-19 (Q28) were asked. We assumed the respondent was COVID-19+ (n = 144) if the subject claimed to have been hospitalized or stayed in a hotel for COVID-19 treatment. COVID-19− subjects (n = 8,693) were defined as those who claimed they were not infected with COVID-19 (Fig. 1). Subjects who visited clinics with symptoms or were advised to isolate themselves at home due to close contact with COVID-19, as well as those whose relatives, friends or coworkers were diagnosed as COVID-19+ were excluded from the COVID-19− group. Furthermore, we excluded subjects from the COVID-19− group who were in poor physical condition, such as having persistent fatigue, or flu symptoms (sore throat, cough, sputum, and fever) between the period of January 2020 and February 2021. Because FLU susceptible patients are routinely screened by rapid antigen testing at most general outpatient clinics in Japan and are covered by medical insurance, all claims for FLU positivity were counted as such.

### Statistical analysis

The data analyses were performed using Python and R languages, and participants’ characteristics were summarized using mean (± standard deviation) scores or percentages (frequency counts). Using the methods described in Supplemental Fig. S1, we consolidated the 144 variables to 59. An independent sample Mann Whitney U test (Kruskal–Wallis test for 3 groups) or chi-square was conducted to investigate potential differences in sociodemographics, and sleep variables of participants with COVID+19+ vs. COVID-19−, SAS+ vs. SAS−, and FLU+ vs. FLU− (The results of univariate analysis for COVID+19+ vs. COVID-19−, SAS+ vs. SAS− are presented in Supplemental Material S4). Multivariable logistic regression analyses with 95% confidence intervals (CI), were then performed with 8 to 12 variables, depending on the number of subjects used in each analysis, to investigate the relationship between the risk of COVID-19, SAS and other variables (Fig. S1, Tables 6, 7 Tables S3-1 and S3-2). The variables for the models were chosen based on statistical significance and clinical importance. Namely, “oversleep” and “sleep longer during weekend” since chronic sleep loss is one of the most prominent sleep characteristics of Japanese workers, since chronic sleep loss is known to relate to reduction in immune functions and metabolic diseases. A p-value of less than 0.05 was considered statistically significant (2-sided).

## Supplementary Information

Supplementary Information.

## Data Availability

The datasets used and/or analyzed during the current study available from the corresponding author on reasonable request.

## Abbreviations

SAS: Sleep apnea syndrome
OSA: Obstructive sleep apnea
BMI: Body mass index
PSQI: Pittsburgh Sleep Quality Index
PPI: Proton pump inhibitors
GERD: Gastroesophageal reflux disease
