# Supplementary material for: Influences of sleep and lifestyle factors on the risk for covid-19 infections, from internet survey of 10,000 Japanese business workers
Source: Sci Rep. 2022 Nov 16;12:19640. doi: 10.1038/s41598-022-22105-3 (PMC9666950; doi:10.1038/s41598-022-22105-3)
Supplement: Supplementary file 1 — Supplementary Information. [file 41598_2022_22105_MOESM1_ESM.docx]

**Supplementary information:**

Summary tables of the anthropometric, demographic variables (S1 [1-4])

Data Cleansing Flow Chart (Fig. S1)

Data Cleansing Flow Tables (Table. S2)

Table S3-1, S3-2

Table S4(1-8)

Table S5. Survey questionnaires

**Table S1-1. Distribution by weight**

|  | **Weight [kg]** | | | | | | | | | | |  |
| --- | --- | --- | --- | --- | --- | --- | --- | --- | --- | --- | --- | --- |
| **Sex** | **-40** | **40-45** | **45-50** | **50-55** | **55-60** | **60-65** | **65-70** | **70-75** | **75-80** | **40-45** | **45-50** | **Sum** |
| Male | 11 | 25 | 85 | 318 | 574 | 1,029 | 966 | 816 | 488 | 382 | 465 | 5,159 |
| (%) | 0.2% | 0.5% | 1.6% | 6.2% | 11.1% | 19.9% | 18.7% | 15.8% | 9.5% | 7.4% | 9.0% | 100.0% |
| Female | 119 | 533 | 1,203 | 1,363 | 857 | 525 | 253 | 147 | 67 | 51 | 46 | 5,164 |
| (%) | 2.3% | 10.3% | 23.3% | 26.4% | 16.6% | 10.2% | 4.9% | 2.8% | 1.3% | 1.0% | 0.9% | 100.0% |
| **Total** | **130** | **558** | **1,288** | **1,681** | **1,431** | **1,554** | **1,219** | **963** | **555** | **433** | **511** | **10,323** |
| **(%)** | 1.3% | 5.4% | 12.5% | 16.3% | 13.9% | 15.1% | 11.8% | 9.3% | 5.4% | 4.2% | 5.0% | 100.0% |

**Table S1-2. Distribution by height**

|  | **Height [m]** | | | | | | | | | | |  |
| --- | --- | --- | --- | --- | --- | --- | --- | --- | --- | --- | --- | --- |
| **Sex** | **-1.45** | **1.45-1.50** | **1.50-1.55** | **1.55-1.60** | **1.60-1.65** | **1.65-1.70** | **1.70-1.75** | **1.75-1.80** | **1.80-1.85** | **1.85-1.90** | **1.90-** | **Sum** |
| Male | 2 | 0 | 12 | 78 | 493 | 1,276 | 1,798 | 1,039 | 386 | 63 | 12 | 5,159 |
| (%) | 0.0% | 0.0% | 0.2% | 1.5% | 9.6% | 24.7% | 34.9% | 20.1% | 7.5% | 1.2% | 0.2% | 100.0% |
| Female | 20 | 196 | 1,077 | 1,739 | 1,473 | 523 | 113 | 21 | 1 | 0 | 1 | 5,164 |
| (%) | 0.4% | 3.8% | 20.9% | 33.7% | 28.5% | 10.1% | 2.2% | 0.4% | 0.0% | 0.0% | 0.0% | 100.0% |
| **Total** | **22** | **196** | **1,089** | **1,817** | **1,966** | **1,799** | **1,911** | **1,060** | **387** | **63** | **13** | **10,323** |
| **(%)** | 0.2% | 1.9% | 10.5% | 17.6% | 19.0% | 17.4% | 18.5% | 10.3% | 3.7% | 0.6% | 0.1% | 100.0% |

**Table S1-3. Distribution by BMI**

|  | **BMI** | | | | | | | | | | |  |
| --- | --- | --- | --- | --- | --- | --- | --- | --- | --- | --- | --- | --- |
| **Sex** | **-17.0** | **17.0-18.5** | **18.5-20.0** | **20.0-21.5** | **21.5-23.0** | **23.0-24.5** | **24.5-26.0** | **26.0-27.5** | **27.5-29.0** | **29.0-30.5** | **30.5-** | **Sum** |
| Male | 80 | 226 | 503 | 935 | 1,019 | 826 | 562 | 389 | 227 | 204 | 188 | 5,159 |
| (%) | 1.6% | 4.4% | 9.7% | 18.1% | 19.8% | 16.0% | 10.9% | 7.5% | 4.4% | 4.0% | 3.6% | 100.0% |
| Female | 255 | 742 | 1,069 | 1,104 | 766 | 498 | 274 | 178 | 89 | 82 | 107 | 5,164 |
| (%) | 4.9% | 14.4% | 20.7% | 21.4% | 14.8% | 9.6% | 5.3% | 3.4% | 1.7% | 1.6% | 2.1% | 100.0% |
| **Total** | **335** | **968** | **1,572** | **2,039** | **1,785** | **1,324** | **836** | **567** | **316** | **286** | **295** | **10,323** |
| **(%)** | 3.2% | 9.4% | 15.2% | 19.8% | 17.3% | 12.8% | 8.1% | 5.5% | 3.1% | 2.8% | 2.9% | 100.0% |

**Table S1-4. Distribution by prefecture, sex and ages**

|  |  | **Age** | | |  | **Total** |  |  |  | **Age** | | |  | **Total** |
| --- | --- | --- | --- | --- | --- | --- | --- | --- | --- | --- | --- | --- | --- | --- |
| **Prefecture** | **Sex** | **20-29** | **30-49** | **50-** | **Sum** | **Sum** |  | **Prefecture** | **Sex** | **20-29** | **30-49** | **50-** | **Sum** | **Sum** |
| **Hokkaido** | Male | 31 | 52 | 52 | 135 | **280** |  | **Shiga** | Male | 31 | 31 | 31 | 93 | **184** |
|  | Female | 31 | 52 | 62 | 145 |  |  |  | Female | 30 | 30 | 31 | 91 |  |
| **Aomori** | Male | 31 | 31 | 31 | 93 | **186** |  | **Kyoto** | Male | 31 | 31 | 31 | 93 | **185** |
|  | Female | 31 | 31 | 31 | 93 |  |  |  | Female | 30 | 31 | 31 | 92 |  |
| **Iwate** | Male | 31 | 31 | 31 | 93 | **186** |  | **Osaka** | Male | 31 | 83 | 83 | 197 | **394** |
|  | Female | 31 | 31 | 31 | 93 |  |  |  | Female | 31 | 83 | 83 | 197 |  |
| **Miyagi** | Male | 31 | 31 | 31 | 93 | **186** |  | **Hyogo** | Male | 31 | 52 | 52 | 135 | **270** |
|  | Female | 31 | 31 | 31 | 93 |  |  |  | Female | 31 | 52 | 52 | 135 |  |
| **Akita** | Male | 30 | 31 | 31 | 92 | **184** |  | **Nara** | Male | 31 | 31 | 31 | 93 | **186** |
|  | Female | 31 | 31 | 30 | 92 |  |  |  | Female | 31 | 31 | 31 | 93 |  |
| **Yamagata** | Male | 31 | 31 | 31 | 93 | **186** |  | **Wakayama** | Male | 31 | 31 | 31 | 93 | **186** |
|  | Female | 31 | 31 | 31 | 93 |  |  |  | Female | 31 | 31 | 31 | 93 |  |
| **Fukushima** | Male | 31 | 31 | 31 | 93 | **186** |  | **Tottori** | Male | 27 | 31 | 31 | 89 | **182** |
|  | Female | 31 | 31 | 31 | 93 |  |  |  | Female | 31 | 31 | 31 | 93 |  |
| **Ibaraki** | Male | 31 | 31 | 31 | 93 | **186** |  | **Shimane** | Male | 31 | 30 | 31 | 92 | **185** |
|  | Female | 31 | 31 | 31 | 93 |  |  |  | Female | 31 | 31 | 31 | 93 |  |
| **Tochigi** | Male | 31 | 31 | 31 | 93 | **186** |  | **Okayama** | Male | 31 | 31 | 31 | 93 | **186** |
|  | Female | 31 | 31 | 31 | 93 |  |  |  | Female | 31 | 31 | 31 | 93 |  |
| **Gunma** | Male | 31 | 31 | 31 | 93 | **186** |  | **Hiroshima** | Male | 31 | 31 | 31 | 93 | **186** |
|  | Female | 31 | 31 | 31 | 93 |  |  |  | Female | 31 | 31 | 31 | 93 |  |
| **Saitama** | Male | 30 | 73 | 73 | 176 | **353** |  | **Yamaguchi** | Male | 31 | 31 | 31 | 93 | **186** |
|  | Female | 31 | 73 | 73 | 177 |  |  |  | Female | 31 | 31 | 31 | 93 |  |
| **Chiba** | Male | 31 | 62 | 62 | 155 | **310** |  | **Tokushima** | Male | 31 | 31 | 31 | 93 | **186** |
|  | Female | 31 | 62 | 62 | 155 |  |  |  | Female | 31 | 31 | 31 | 93 |  |
| **Tokyo** | Male | 62 | 155 | 114 | 331 | **650** |  | **Kagawa** | Male | 31 | 31 | 31 | 93 | **186** |
|  | Female | 61 | 155 | 103 | 319 |  |  |  | Female | 31 | 31 | 31 | 93 |  |
| **Kanagawa** | Male | 31 | 93 | 82 | 206 | **412** |  | **Ehime** | Male | 31 | 31 | 31 | 93 | **186** |
|  | Female | 31 | 92 | 83 | 206 |  |  |  | Female | 31 | 31 | 31 | 93 |  |
| **Niigata** | Male | 31 | 31 | 31 | 93 | **186** |  | **Kochi** | Male | 31 | 31 | 31 | 93 | **186** |
|  | Female | 31 | 31 | 31 | 93 |  |  |  | Female | 31 | 31 | 31 | 93 |  |
| **Toyama** | Male | 31 | 31 | 31 | 93 | **186** |  | **Fukuoka** | Male | 31 | 52 | 52 | 135 | **270** |
|  | Female | 31 | 31 | 31 | 93 |  |  |  | Female | 31 | 52 | 52 | 135 |  |
| **Ishikawa** | Male | 31 | 31 | 31 | 93 | **186** |  | **Saga** | Male | 31 | 31 | 31 | 93 | **186** |
|  | Female | 31 | 31 | 31 | 93 |  |  |  | Female | 31 | 31 | 31 | 93 |  |
| **Fukui** | Male | 31 | 31 | 31 | 93 | **186** |  | **Nagasaki** | Male | 31 | 31 | 31 | 93 | **186** |
|  | Female | 31 | 31 | 31 | 93 |  |  |  | Female | 31 | 31 | 31 | 93 |  |
| **Yamanashi** | Male | 31 | 31 | 31 | 93 | **186** |  | **Kumamoto** | Male | 31 | 31 | 31 | 93 | **186** |
|  | Female | 31 | 31 | 31 | 93 |  |  |  | Female | 31 | 31 | 31 | 93 |  |
| **Nagano** | Male | 31 | 31 | 31 | 93 | **186** |  | **Oita** | Male | 31 | 30 | 31 | 92 | **185** |
|  | Female | 31 | 31 | 31 | 93 |  |  |  | Female | 31 | 31 | 31 | 93 |  |
| **Gifu** | Male | 31 | 31 | 31 | 93 | **186** |  | **Miyazaki** | Male | 29 | 31 | 31 | 91 | **184** |
|  | Female | 31 | 31 | 31 | 93 |  |  |  | Female | 31 | 31 | 31 | 93 |  |
| **Shizuoka** | Male | 31 | 31 | 31 | 93 | **185** |  | **Kagoshima** | Male | 31 | 30 | 31 | 92 | **185** |
|  | Female | 30 | 31 | 31 | 92 |  |  |  | Female | 31 | 31 | 31 | 93 |  |
| **Aichi** | Male | 31 | 72 | 62 | 165 | **331** |  | **Okinawa** | Male | 31 | 31 | 31 | 93 | **186** |
|  | Female | 31 | 73 | 62 | 166 |  |  |  | Female | 31 | 31 | 31 | 93 |  |
| **Mie** | Male | 31 | 31 | 31 | 93 | **186** |  | **Total** | **Male** | **1,480** | **1,869** | **1,810** | **5,159** | **10,323** |
|  | Female | 31 | 31 | 31 | 93 |  |  |  | **Female** | **1,484** | **1,871** | **1,809** | **5,164** |  |

Data Cleansing Flow Chart (Fig. 1s)


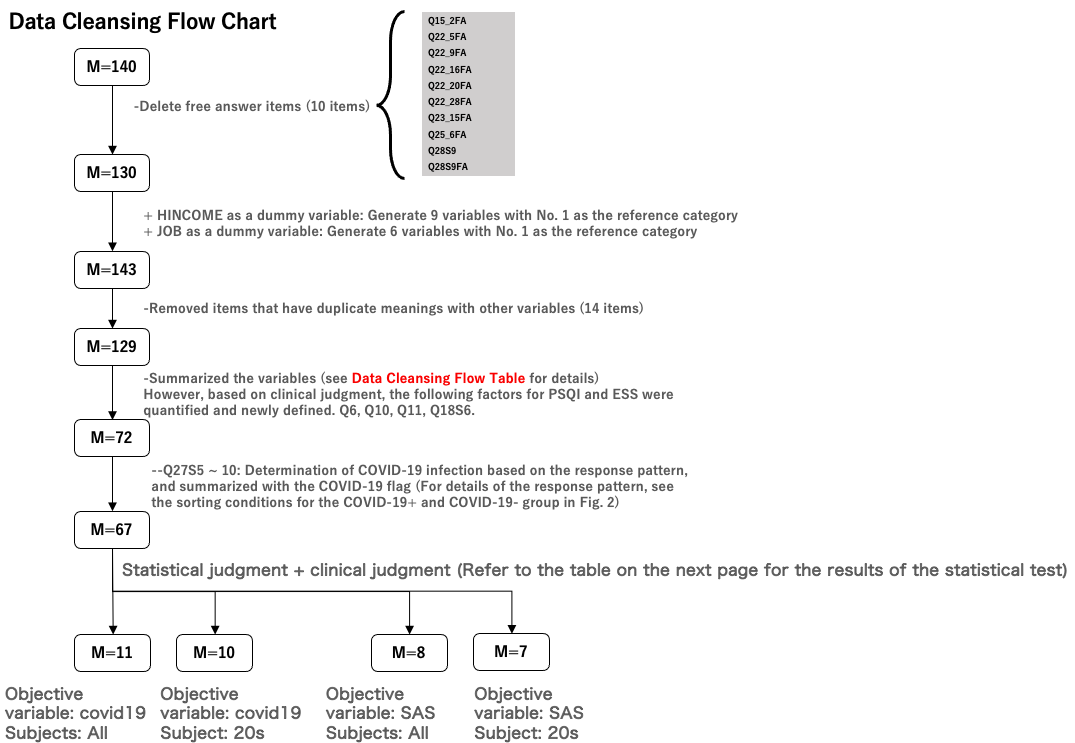


**S2. Data Cleansing Flow Tables**

| **#** | **Variables after summarization** | **Variables which were the source of summarization** | **Overview of summarization** |  |
| --- | --- | --- | --- | --- |
| **1** | **SEX** |  |  | **No Operation** |
| **2** | **AGE** |  |  |  |
| **3** | **MARRIED** |  |  |  |
| **4** | **CHILD** |  |  |  |
| **5** | **Q1S1** |  |  |  |
| **6** | **Q1S2** |  |  |  |
| **7** | **Q1S3** |  |  |  |
| **8** | **Q1S4** |  |  |  |
| **9** | **Q1S5** |  |  |  |
| **10** | **Q1S6** |  |  |  |
| **11** | **Q1S7** |  |  |  |
| **12** | **Q1S8** |  |  |  |
| **13** | **Q13S1** |  |  |  |
| **14** | **Q13S2** |  |  |  |
| **15** | **Q13S3** |  |  |  |
| **16** | **Q13S4** |  |  |  |
| **17** | **Q13S5** |  |  |  |
| **18** | **Q13S6** |  |  |  |
| **19** | **Q13S7** |  |  |  |
| **20** | **Q13S8** |  |  |  |
| **21** | **Q14** |  |  |  |
| **22** | **Q15_1** |  |  |  |
| **23** | **Q15_2** |  |  |  |
| **24** | **Q21S1N** |  |  |  |
| **25** | **Q24** |  |  |  |
| **26** | **Q27S1** |  |  |  |
| **27** | **Q27S2** |  |  |  |
| **28** | **Q27S3** |  |  |  |
| **29** | **Q27S4** |  |  |  |
| **30** | **Q27S5** |  |  |  |
| **31** | **Q27S6** |  |  |  |
| **32** | **Q27S7** |  |  |  |
| **33** | **Q27S8** |  |  |  |
| **34** | **Q27S9** |  |  |  |
| **35** | **Q27S10** |  |  |  |
| **36** | **Q28S1** |  |  |  |
| **37** | **Q28S2** |  |  |  |
| **38** | **Q28S3** |  |  |  |
| **39** | **Q28S4** |  |  |  |
| **40** | **Q28S5** |  |  |  |
| **41** | **Q28S6** |  |  |  |
| **42** | **Q28S7** |  |  |  |
| **43** | **Q28S8** |  |  |  |

S2. Data Cleansing Flow Table (continued)

| **#** | **Variables after summarization** | **Variables which were the source of summarization** | **Overview of**  **summarization** |  |
| --- | --- | --- | --- | --- |
| **44** | **Q16_global_score** | **Q16S1N-Q16S4N** | **Total value of the left box** | **Summarized** |
| **45** | **Q17_global_score** | **Q17S1N/Q17S2N** | **Product of the left box** |  |
| **46** | **Q19_global_score** | **Q19S1N/Q19S2N** | **Total value of the left box** |  |
| **47** | **Q20_global_score** | **Q20S1-Q20S29** | **Total value of the left box (Q20S1-S3 had inverted scoring)** |  |
| **48** | **PSQI_global_score** | **Q8S1-Q8S10/Q9/Q10/Q11/Q12** | **Refer to PSQI calculation methods** |  |
| **49** | **ESS_global_score** | **Q18S1-Q18S8** | **Refer to ESS calculation methods** |  |
| **50** | **BMI** | **Q26S1N/Q26S2N** | **Q26S2N/(0.01*Q26S1N)^2** |  |
| **51** | **BrinkmanIndex** | **Q3S1N/Q4S1N** | **Product of the left box** |  |
| **52** | **subjective_sleep_latency** | **Q7S3N** | **Unchanged** |  |
| **53** | **subjective_sleep_duration** | **Q7S1N-Q7S7N** | **Time of falling asleep, time of waking up, and subjective sleep time were asked, but only the subjective sleep time was used (the chronotype was discarded).** |  |
| **54** | **Q6_binary** | **Q6** | **123 to 0, 456 to1** | **Binarization** |
| **55** | **Q10_binary** | **Q10** | **12 to 0,34 to 1** |  |
| **56** | **Q11_binary** | **Q11** | **12 to 0,34 to 1** |  |
| **57** | **Q18S6_binaray** | **Q18S6** | **12 to 0,34 to 1** |  |
| **58** | **HINCOME_2** | **HINCOME** | **1 as a reference** | **Dummy variable** |
| **59** | **HINCOME_3** | **HINCOME** | **1 as a reference** |  |
| **60** | **HINCOME_4** | **HINCOME** | **1 as a reference** |  |
| **61** | **HINCOME_5** | **HINCOME** | **1 as a reference** |  |
| **62** | **HINCOME_6** | **HINCOME** | **1 as a reference** |  |
| **63** | **HINCOME_7** | **HINCOME** | **1 as a reference** |  |
| **64** | **HINCOME_8** | **HINCOME** | **1 as a reference** |  |
| **65** | **HINCOME_9** | **HINCOME** | **1 as a reference** |  |
| **66** | **HINCOME_10** | **HINCOME** | **1 as a reference** |  |
| **67** | **JOB_2** | **JOB** | **1 as a reference** |  |
| **68** | **JOB_3** | **JOB** | **1 as a reference** |  |
| **69** | **JOB_4** | **JOB** | **1 as a reference** |  |
| **70** | **JOB_5** | **JOB** | **1 as a reference** |  |
| **71** | **JOB_6** | **JOB** | **1 as a reference** |  |
| **72** | **JOB_7** | **JOB** | **1 as a reference** |  |

| **Table S3-1. Odd ratio and 95% confidence interval for the significant association of the variables with COVID-19+, 20s** | | | | | | | | |
| --- | --- | --- | --- | --- | --- | --- | --- | --- |
|  |  |  |  | **95%  confidence interval** | |  |  |  |
| **Variables** | **coeff** | **z value** | **OR** | **lower** | **upper** | **p value** | **Question ID** |  |
| Going out without face mask | 2.45 | 7.90 | 11.57 | 6.30 | 21.20 | **2.73E-15** | Q28S5 |  |
| FLU+ | 1.87 | 5.15 | 6.48 | 3.18 | 13.20 | **2.58E-07** | Q27S4 |  |
| SAS+ | 1.99 | 5.15 | 7.34 | 3.44 | 15.68 | **2.62E-07** | Q15_1 |  |
| Excessive exercise before going to bed | 0.76 | 0.47 | 2.13 | 1.51 | 2.99 | **1.29E-05** | Q1S1 |  |
| Falling asleep while sitting or talking with someone | 1.29 | 0.27 | 3.64 | 1.94 | 6.83 | **5.51E-05** | Q18S6_binary |  |
| Use of hypnotics | 1.18 | 2.45 | 3.25 | 1.27 | 8.32 | **0.014** | Q10_binary |  |
| SEX (male) | 0.65 | 0.52 | 1.92 | 1.01 | 3.65 | **0.048** | SEX |  |
| Sleep longer during weekend | 0.38 | 1.27 | 1.47 | 0.81 | 2.65 | 0.21 | Q6_binary |  |
| Sever sleepiness while driving | 0.24 | 0.56 | 1.27 | 0.34 | 1.81 | 0.57 | Q11_binary |  |
| BMI | 0.01 | 0.99 | 1.01 | 0.95 | 1.08 | 0.69 | BMI |  |
| Oversleep (Getting up late) | 0.01 | 0.07 | 1.01 | 0.69 | 1.41 | 0.94 | Q1S6 |  |

| **Table S3-2. Odd ratio and 95% confidence interval for the significant association of the variables with SAS+, 20s** | | | | | | | | |
| --- | --- | --- | --- | --- | --- | --- | --- | --- |
|  |  |  |  | **95%  confidence interval** | |  |  |  |
| **Variables** | **coeff** | **z value** | **OR** | **lower** | **upper** | **p value** | **Question ID** |  |
| COVID-19+ | 2.39 | 6.54 | 10.90 | 5.33 | 22.31 | **6.09E-11** | covid19_flag |  |
| Self-reported as a short sleeper | 1.82 | 5.73 | 6.15 | 3.30 | 11.44 | **9.83E-09** | Q14 |  |
| Work performance | 0.01 | 0.99 | 1.01 | 1.00 | 1.01 | **0.00057** | Q16_global  _score |  |
| Remote work (working from home) | 0.28 | 0.76 | 1.32 | 1.09 | 1.60 | **0.0039** | Q24 |  |
| Sleep in a room with the TV/light on | 0.48 | 0.62 | 1.61 | 1.16 | 2.23 | **0.0041** | Q1S4 |  |
| Sleep right after eating a meal | 0.37 | 0.69 | 1.45 | 1.10 | 1.92 | **0.0082** | Q1S3 |  |
| BMI | 0.07 | 1.98 | 1.07 | 1.00 | 1.14 | **0.048** | BMI |  |
| SEX (male) | 0.63 | 0.53 | 1.88 | 0.95 | 3.72 | 0.069 | SEX |  |

| **S4-1. Chi Squire analysis for COVID-19 positivity for all age** | | |  |  |
| --- | --- | --- | --- | --- |
| **Variables** | **x2** | **p value** | **dof** | **Question ID** |
| covid19_flag | 8774.73 | 0 | 1 | covid19_flag |
| Falling asleep while sitting or talking with someone | 537.49 | 6.62E-119 | 1 | Q18S6_binary |
| SAS+ | 481.54 | 9.90E-107 | 1 | Q15_1 |
| FLU+ | 422.68 | 6.37E-94 | 1 | Q27S4 |
| Going out without face mask | 222.10 | 3.15E-50 | 1 | Q28S5 |
| Use of hypnotics | 171.42 | 3.62E-39 | 1 | Q10_binary |
| Severe sleepiness while driving, eating meals, engaging in social activity | 141.77 | 1.09E-32 | 1 | Q11_binary |
| Have you realized or been told by anyone of stopped breathing while sleeping | 138.38 | 6.00E-32 | 1 | Q13S2 |
| Avoid places where people gather | 67.94 | 1.69E-16 | 1 | Q28S2 |
| Avoid places with poor ventilation | 64.05 | 1.21E-15 | 1 | Q28S1 |
| Disinfect with hand washing, gargling and/or alcohol | 59.35 | 1.32E-14 | 1 | Q28S4 |
| Avoid unnecessary and non-essential outings | 55.66 | 8.61E-14 | 1 | Q28S6 |
| I have been telecommuting | 52.79 | 3.70E-13 | 1 | Q28S7 |
| Do you recently find yourself falling asleep before you realize? | 48.59 | 3.15E-12 | 1 | Q13S3 |
| SEX | 35.66 | 2.34E-09 | 1 | SEX |
| Relatives having any of the followings: a) cardiovascular/cerebrovascular diseases, b) high blood pressure, c) hyperlipemia, or d) diabetes? | 30.90 | 2.72E-08 | 1 | Q13S8 |
| Do you recognize yourself as a short sleeper | 29.76 | 4.89E-08 | 1 | Q14 |
| I experienced cold symptoms | 20.87 | 4.92E-06 | 1 | Q27S2 |
| Antibacterial goods | 20.41 | 6.25E-06 | 1 | Q28S8 |
| Have you had rhinitis or bad teeth alignment from an early age | 13.68 | 0.0002 | 1 | Q13S7 |
| JOB_4 | 10.48 | 0.0012 | 1 | JOB_4 |
| I have felt some kind of illness | 6.24 | 0.012 | 1 | Q27S1 |
| Conversations with other people in close proximity | 6.03 | 0.014 | 1 | Q28S3 |
| Have you realized or been told by anyone that you snore while sleeping? | 5.79 | 0.016 | 1 | Q13S1 |
| Has your weight increased by more than 10kg compared to when you were 20 years old? | 5.78 | 0.016 | 1 | Q13S6 |
| Sleep longer during weekend | 5.37 | 0.021 | 1 | Q6_binary |
| JOB_6 | 5.34 | 0.021 | 1 | JOB_6 |
| I received the influenza vaccine | 4.07 | 0.044 | 1 | Q27S3 |
| HINCOME_9 | 1.93 | 0.16 | 1 | HINCOME_9 |
| CHILD | 1.76 | 0.18 | 1 | CHILD |
| Sleep Disorders | 1.44 | 0.23 | 1 | Q15_2 |
| MARRIED | 1.27 | 0.26 | 1 | MARRIED |
| Do you still feel tired when you wake up? | 1.18 | 0.28 | 1 | Q13S4 |
| HINCOME_8 | 0.94 | 0.33 | 1 | HINCOME_8 |
| HINCOME_6 | 0.87 | 0.35 | 1 | HINCOME_6 |
| HINCOME_7 | 0.32 | 0.57 | 1 | HINCOME_7 |
| HINCOME_3 | 0.23 | 0.63 | 1 | HINCOME_3 |
| HINCOME_10 | 0.22 | 0.64 | 1 | HINCOME_10 |
| JOB_5 | 0.13 | 0.72 | 1 | JOB_5 |
| Falling asleep before you realize | 0.10 | 0.75 | 1 | Q13S3 |
| JOB_2 | 0.04 | 0.84 | 1 | JOB_2 |
| JOB_3 | 0.02 | 0.89 | 1 | JOB_3 |
| JOB_7 | 0.00 | 0.99 | 1 | JOB_7 |
| HINCOME_5 | 0.00 | 0.99 | 1 | HINCOME_5 |
| HINCOME_2 | 0.00 | 0.99 | 1 | HINCOME_2 |
| HINCOME_4 | 0.00 | 0.99 | 1 | HINCOME_4 |

| **S4-2. Mann-Whitney analysis for COVID-19 positivity for all age** | | |  |  |
| --- | --- | --- | --- | --- |
| **Variables** | **p value** | **Mann-Whitney  U-stat** | **COVID-19+ >COVID-19-** | **Question ID** |
| Excessive exercise before going to bed | 5.12E-126 | 927880 | TRUE | Q1S1 |
| ESS_global_score | 7.30E-24 | 320672 | FALSE | ESS_global_score |
| Oversleep | 5.86E-23 | 834634.5 | TRUE | Q1S6 |
| Sleep right after eating a meal | 7.75E-23 | 846827.5 | TRUE | Q1S3 |
| AGE | 2.02E-21 | 914353 | TRUE | AGE |
| Too sleepy at work | 2.72E-20 | 392513 | FALSE | Q17_global_score |
| Drink alcohol before bedtime | 5.75E-18 | 840881 | TRUE | Q1S8 |
| Q20_global_score | 8.17E-18 | 364906.5 | FALSE | Q20_global_score |
| Sleep in a room with the TV/light on | 3.11E-17 | 816509.5 | TRUE | Q1S4 |
| PSQI_global_score | 1.11E-13 | 402229 | FALSE | PSQI_global_score |
| Work performance | 2.29E-13 | 848291.5 | TRUE | Q16_global_score |
| Main working situation | 2.88E-13 | 806742.5 | TRUE | Q24 |
| Take a bath within 90 minutes before bed | 1.27E-09 | 796538.5 | TRUE | Q1S7 |
| View the screen of your computer, smartphone or tablet before going to bed | 2.55E-08 | 493422 | FALSE | Q1S2 |
| Melancholy | 8.32E-08 | 469769 | FALSE | Q21S1N |
| Go to bed at different times | 5.20E-05 | 742608.5 | TRUE | Q1S5 |
| BrinkmanIndex | 0.00045809 | 551394.5 | TRUE | BrinkmanIndex |
| Working hours | 0.001252471 | 529718.5 | FALSE | Q19_global_score |
| subjective_sleep_duration | 0.419105576 | 601591.5 | FALSE | subjective_sleep  _duration |
| subjective_sleep_latency | 0.587147154 | 642127.5 | TRUE | subjective_sleep  _latency |
| BMI | 0.900639132 | 622104.5 | TRUE | BMI |

| **S4-3. Chi Squire analysis for SAS positivity for all age** |  |  |  |  |
| --- | --- | --- | --- | --- |
| **Variables** | **x2** | **p value** | **dof** | **Question ID** |
| SAS+ | 8804.7 | 0 | 1 | Q15_1 |
| Have you realized or been told by anyone of stopped breathing while sleeping | 1234.6 | 1.85E-270 | 1 | Q13S2 |
| covid19_flag | 481.5 | 9.90E-107 | 1 | covid19_flag |
| Have you realized or been told by anyone that you snore while sleeping? | 187.5 | 1.14E-42 | 1 | Q13S1 |
| Use of hypnotics | 158.1 | 2.87E-36 | 1 | Q10_binary |
| Falling asleep while sitting or talking with someone | 157.8 | 3.37E-36 | 1 | Q18S6_binary |
| Has your weight increased by more than 10kg compared to when you were 20 years old? | 151.2 | 9.40E-35 | 1 | Q13S6 |
| SEX | 142.3 | 8.52E-33 | 1 | SEX |
| Relatives having any of the followings: a) cardiovascular/cerebrovascular diseases, b) high blood pressure, c) hyperlipemia, or d) diabetes? | 105.6 | 8.84E-25 | 1 | Q13S8 |
| FLU+ | 105.4 | 1.01E-24 | 1 | Q27S4 |
| Severe sleepiness while driving, eating meals, engaging in social activity | 84.4 | 4.05E-20 | 1 | Q11_binary |
| Falling asleep before you realize | 78.9 | 6.38E-19 | 1 | Q13S3 |
| Do you recognize yourself as a short sleeper | 70.8 | 3.86E-17 | 1 | Q14 |
| I have been telecommuting | 64.1 | 1.20E-15 | 1 | Q28S7 |
| Have you had rhinitis or bad teeth alignment from an early age | 36.4 | 1.57E-09 | 1 | Q13S7 |
| CHILD | 21.8 | 2.95E-06 | 1 | CHILD |
| Going out without face mask | 17.2 | 3.28E-05 | 1 | Q28S5 |
| Recently have you had to wake up at night to use the bathroom? | 16.5 | 4.81E-05 | 1 | Q13S5 |
| Antibacterial goods | 15.7 | 7.49E-05 | 1 | Q28S8 |
| I experienced cold symptoms | 14.3 | 0.00015 | 1 | Q27S2 |
| HINCOME_10 | 13.9 | 0.00019 | 1 | HINCOME_10 |
| MARRIED | 12.6 | 0.00038 | 1 | MARRIED |
| I experienced cold symptoms | 11.7 | 0.00062 | 1 | Q27S3 |
| Do you still feel tired when you wake up? | 10.0 | 0.0016 | 1 | Q13S4 |
| JOB_4 | 8.8 | 0.0031 | 1 | JOB_4 |
| I have felt some kind of illness | 8.0 | 0.0047 | 1 | Q27S1 |
| JOB_2 | 6.9 | 0.0086 | 1 | JOB_2 |
| JOB_5 | 4.3 | 0.037 | 1 | JOB_5 |
| HINCOME_6 | 3.5 | 0.062 | 1 | HINCOME_6 |
| HINCOME_7 | 3.4 | 0.063 | 1 | HINCOME_7 |
| JOB_3 | 2.3 | 0.13 | 1 | JOB_3 |
| Sleep longer during weekend | 2.2 | 0.14 | 1 | Q6_binary |
| Avoid unnecessary and non-essential outings | 1.7 | 0.20 | 1 | Q28S6 |
| Conversations with other people in close proximity | 1.5 | 0.22 | 1 | Q28S3 |
| HINCOME_3 | 1.5 | 0.22 | 1 | HINCOME_3 |
| HINCOME_9 | 1.0 | 0.33 | 1 | HINCOME_9 |
| Sleep Disorders | 0.9 | 0.34 | 1 | Q15_2 |
| HINCOME_4 | 0.8 | 0.36 | 1 | HINCOME_4 |
| HINCOME_5 | 0.7 | 0.39 | 1 | HINCOME_5 |
| Avoid places where people gather | 0.7 | 0.42 | 1 | Q28S2 |
| Disinfect with hand washing, gargling and/or alcohol28S4 | 0.6 | 0.42 | 1 | Q28S4 |
| HINCOME_8 | 0.3 | 0.59 | 1 | HINCOME_8 |
| Avoid places with poor ventilation | 0.2 | 0.62 | 1 | Q28S1 |
| HINCOME_2 | 0.2 | 0.62 | 1 | HINCOME_2 |
| JOB_7 | 0.1 | 0.79 | 1 | JOB_7 |
| JOB_6 | 0.0 | 0.87 | 1 | JOB_6 |

| **S4-4. Mann-Whitney analysis for SAS positivity for all age** | |  |  |  |
| --- | --- | --- | --- | --- |
| **Variables** | **p value** | **Mann-Whitney  U-stat** | **SAS+>SAS-** | **Question ID** |
| Excessive exercise before going to bed | 7.93E-42 | 1444148 | TRUE | Q1S1 |
| BMI | 5.19E-26 | 761634 | FALSE | BMI |
| Sleep right after eating a meal | 9.26E-26 | 1533409 | TRUE | Q1S3 |
| Too sleepy at work | 5.75E-21 | 876461 | FALSE | Q17_global_score |
| PSQI_global_score | 5.27E-20 | 823365 | FALSE | PSQI_global_score |
| Drink alcohol before bedtime | 2.03E-19 | 1517634 | TRUE | Q1S8 |
| ESS_global_score | 1.28E-17 | 846764 | FALSE | ESS_global_score |
| Main working situation | 3.48E-17 | 1496153 | TRUE | Q24 |
| Work performance | 1.75E-13 | 1516510 | TRUE | Q16_global_score |
| Sleep in a room with the TV/light on | 3.05E-13 | 1434828 | TRUE | Q1S4 |
| BrinkmanIndex | 7.29E-13 | 994516 | FALSE | BrinkmanIndex |
| Oversleep | 3.57E-11 | 1400733 | TRUE | Q1S6 |
| Mood | 1.32E-08 | 966731 | FALSE | Q20_global_score |
| Go to bed at different times | 6.31E-07 | 1405715.5 | TRUE | Q1S5 |
| Working hours | 1.72E-06 | 1008315.5 | FALSE | Q19_global_score |
| Melancholy | 9.97E-05 | 1048899.5 | FALSE | Q21S1N |
| AGE | 0.001448031 | 1072055.5 | FALSE | AGE |
| Take a bath within 90 minutes before bed | 0.004648226 | 1316701 | TRUE | Q1S7 |
| View the screen of your computer, smartphone or tablet before going to bed | 0.077850225 | 1148030.5 | FALSE | Q1S2 |
| subjective_sleep_duration | 0.251316686 | 1254159.5 | TRUE | subjective_sleep  _duration |
| subjective_sleep_latency | 0.843200322 | 1214464 | TRUE | subjective_sleep  _latency |

| **S4-5. Chi Squire analysis for COVID positivity in 20's** |  |  |  |  |
| --- | --- | --- | --- | --- |
| **Variables** | **x2** | **p value** | **dof** | **Question ID** |
| covid19_flag | 2335.69 | 0 | 1 | covid19_flag |
| SAS+ | 489.42 | 1.9082E-108 | 1 | Q15_1 |
| Falling asleep while sitting or talking with someone | 247.67 | 8.3579E-56 | 1 | Q18S6_binary |
| FLU+ | 246.59 | 1.4408E-55 | 1 | Q27S4 |
| Have you realized or been told by anyone of stopped breathing while sleeping | 224.90 | 7.70477E-51 | 1 | Q13S2 |
| Going out without face mask | 209.29 | 1.95828E-47 | 1 | Q28S5 |
| Use of hypnotics | 205.63 | 1.23182E-46 | 1 | Q10_binary |
| Severe sleepiness while driving, eating meals, engaging in social activity | 94.41 | 2.56515E-22 | 1 | Q11_binary |
| Do you recognize yourself as a short sleeper | 56.75 | 4.94916E-14 | 1 | Q14 |
| Avoid places where people gather | 47.99 | 4.27881E-12 | 1 | Q28S2 |
| Relatives having any of the followings: a) cardiovascular/cerebrovascular diseases, b) high blood pressure, c) hyperlipemia, or d) diabetes? | 46.49 | 9.23161E-12 | 1 | Q13S8 |
| Disinfect with hand washing, gargling and/or alcohol28S4 | 46.13 | 1.10489E-11 | 1 | Q28S4 |
| I have been telecommuting | 45.37 | 1.63005E-11 | 1 | Q28S7 |
| Has your weight increased by more than 10kg compared to when you were 20 years old? | 40.18 | 2.31526E-10 | 1 | Q13S6 |
| SEX | 36.74 | 1.35168E-09 | 1 | SEX |
| Avoid places with poor ventilation | 32.18 | 1.40511E-08 | 1 | Q28S1 |
| Avoid unnecessary and non-essential outings | 27.27 | 1.76839E-07 | 1 | Q28S6 |
| Do you recently find yourself falling asleep before you realize? | 23.86 | 1.03763E-06 | 1 | Q13S3 |
| Antibacterial goods | 18.74 | 1.49479E-05 | 1 | Q28S8 |
| Have you realized or been told by anyone that you snore while sleeping? | 17.83 | 2.4093E-05 | 1 | Q13S1 |
| I experienced cold symptoms | 12.18 | 0.00048 | 1 | Q27S2 |
| HINCOME_9 | 8.56 | 0.0034 | 1 | HINCOME_9 |
| Have you had rhinitis or bad teeth alignment from an early age | 8.24 | 0.0041 | 1 | Q13S7 |
| I have felt some kind of illness | 7.28 | 0.0070 | 1 | Q27S1 |
| CHILD | 7.27 | 0.0070 | 1 | CHILD |
| Recently have you had to wake up at night to use the bathroom? | 5.51 | 0.019 | 1 | Q13S5 |
| HINCOME_10 | 4.60 | 0.032 | 1 | HINCOME_10 |
| HINCOME_6 | 4.55 | 0.033 | 1 | HINCOME_6 |
| Conversations with other people in close proximity | 3.35 | 0.067 | 1 | Q28S3 |
| I received the influenza vaccine | 2.95 | 0.086 | 1 | Q27S3 |
| HINCOME_7 | 2.92 | 0.087 | 1 | HINCOME_7 |
| JOB_7 | 2.55 | 0.11 | 1 | JOB_7 |
| JOB_2 | 2.55 | 0.11 | 1 | JOB_2 |
| JOB_4 | 1.83 | 0.18 | 1 | JOB_4 |
| MARRIED | 1.25 | 0.26 | 1 | MARRIED |
| JOB_5 | 0.93 | 0.33 | 1 | JOB_5 |
| Sleep longer during weekend | 0.42 | 0.52 | 1 | Q6_binary |
| HINCOME_4 | 0.28 | 0.60 | 1 | HINCOME_4 |
| HINCOME_5 | 0.23 | 0.63 | 1 | HINCOME_5 |
| Sleep Disorders | 0.19 | 0.66 | 1 | Q15_2 |
| JOB_6 | 0.12 | 0.73 | 1 | JOB_6 |
| HINCOME_8 | 0.11 | 0.74 | 1 | HINCOME_8 |
| JOB_3 | 0.08 | 0.78 | 1 | JOB_3 |
| HINCOME_2 | 0.05 | 0.82 | 1 | HINCOME_2 |
| HINCOME_3 | 0.04 | 0.84 | 1 | HINCOME_3 |
| Do you still feel tired when you wake up? | 0.00 | 0.96 | 1 | Q13S4 |

| **S4-6. Mann-Whitney anaysis for COVID positibty in 20's** | |  |  |  |
| --- | --- | --- | --- | --- |
| **Variables** | **p value** | **Mann-Whitney  U-stat** | **COVID-19+ >COVID-19-** | **Question ID** |
| Excessive exercise before going to bed | 8.00E-60 | 169414.5 | TRUE | Q1S1 |
| Drinking alcohol before bedtime | 1.54E-24 | 163503 | TRUE | Q1S8 |
| Sleep right after eating a meal | 9.14E-23 | 160525.5 | TRUE | Q1S3 |
| View the screen of your computer, smartphone or tablet before going to bed | 1.22E-19 | 70932.5 | FALSE | Q1S2 |
| Main working situation | 3.49E-19 | 155817 | TRUE | Q24 |
| Sleep in a room with the TV/light on | 1.29E-18 | 153495 | TRUE | Q1S4 |
| Oversleep | 1.01E-15 | 148381 | TRUE | Q1S6 |
| ESS_global_score | 1.23E-14 | 59170.5 | FALSE | ESS_global_score |
| BrinkmanIndex | 6.27E-12 | 81406.5 | FALSE | BrinkmanIndex |
| Too sleepy at work | 2.07E-10 | 72074.5 | FALSE | Q17_global_score |
| PSQI_global_score | 1.16E-09 | 70104 | FALSE | PSQI_global  _score |
| Take a bath within 90 minutes before bed | 1.40E-09 | 146996.5 | TRUE | Q1S7 |
| Work performance | 2.61E-08 | 146373 | TRUE | Q16_global_score |
| Q20_global_score | 3.78E-07 | 76411.5 | FALSE | Q20_global_score |
| subjective_sleep_latency | 0.00030 | 133147 | TRUE | subjective_sleep  _latency |
| Working hours | 0.0032 | 90841 | FALSE | Q19_global_score |
| AGE | 0.0075 | 127186.5 | TRUE | AGE |
| Go to bed at different times | 0.020 | 124511 | TRUE | Q1S5 |
| BMI | 0.036 | 95984 | FALSE | BMI |
| Melancholy | 0.056 | 97498 | FALSE | Q21S1N |
| subjective_sleep_duration | 0.30 | 116495 | TRUE | subjective_sleep  _duration |

| **S4-7. Chi Squire analysis for SAS positivity in 20's** |  |  |  |  |
| --- | --- | --- | --- | --- |
| **Variables** | **x2** | **p value** | **dof** | **Question ID** |
| SAS+ | 2328.60 | 0 | 1 | Q15_1 |
| Have you realized or been told by anyone of stopped breathing while sleeping | 489.42 | 1.91E-108 | 1 | covid19_flag |
| covid19_flag | 298.10 | 8.55E-67 | 1 | Q13S2 |
| Have you realized or been told by anyone that you snore while sleeping? | 180.69 | 3.43E-41 | 1 | Q10_binary |
| Use of hypnotics | 167.18 | 3.06E-38 | 1 | Q27S4 |
| Falling asleep while sitting or talking with someone | 146.46 | 1.03E-33 | 1 | Q18S6_binary |
| Has your weight increased by more than 10kg compared to when you were 20 years old? | 140.91 | 1.68E-32 | 1 | Q14 |
| SEX | 67.81 | 1.80E-16 | 1 | Q13S6 |
| Relatives having any of the followings: a) cardiovascular/cerebrovascular diseases, b) high blood pressure, c) hyperlipemia, or d) diabetes? | 65.83 | 4.93E-16 | 1 | Q11_binary |
| FLU+ | 58.71 | 1.83E-14 | 1 | Q13S1 |
| Severe sleepiness while driving, eating meals, engaging in social activity | 54.14 | 1.86E-13 | 1 | Q28S7 |
| Falling asleep before you realize | 44.91 | 2.06E-11 | 1 | Q13S8 |
| Do you recognize yourself as a short sleeper | 39.66 | 3.03E-10 | 1 | Q28S5 |
| I have been telecommuting | 34.89 | 3.49E-09 | 1 | Q13S3 |
| Have you had rhinitis or bad teeth alignment from an early age | 31.00 | 2.58E-08 | 1 | SEX |
| CHILD | 29.48 | 5.66E-08 | 1 | JOB_2 |
| Going out without face mask | 21.09 | 4.38E-06 | 1 | Q28S8 |
| Recently have you had to wake up at night to use the bathroom? | 20.86 | 4.93E-06 | 1 | CHILD |
| Antibacterial goods | 16.12 | 5.96E-05 | 1 | Q27S2 |
| I experienced cold symptoms | 12.52 | 0.00040 | 1 | Q13S7 |
| HINCOME_10 | 12.23 | 0.00047 | 1 | HINCOME_9 |
| MARRIED | 10.93 | 0.00094 | 1 | HINCOME_10 |
| I experienced cold symptoms | 8.20 | 0.0042 | 1 | Q13S5 |
| Do you still feel tired when you wake up? | 5.36 | 0.021 | 1 | Q28S4 |
| JOB_4 | 5.33 | 0.021 | 1 | Q28S2 |
| I have felt some kind of illness | 4.23 | 0.040 | 1 | HINCOME_6 |
| JOB_2 | 4.07 | 0.044 | 1 | Q28S6 |
| JOB_5 | 3.88 | 0.049 | 1 | JOB_5 |
| HINCOME_6 | 3.82 | 0.051 | 1 | MARRIED |
| HINCOME_7 | 3.33 | 0.068 | 1 | Q27S3 |
| JOB_3 | 2.04 | 0.15 | 1 | JOB_4 |
| Sleep longer during weekend | 2.02 | 0.16 | 1 | HINCOME_5 |
| Avoid unnecessary and non-essential outings | 1.69 | 0.19 | 1 | HINCOME_7 |
| Conversations with other people in close proximity | 1.44 | 0.23 | 1 | HINCOME_4 |
| HINCOME_3 | 1.27 | 0.26 | 1 | HINCOME_2 |
| HINCOME_9 | 1.15 | 0.28 | 1 | Q28S3 |
| Sleep Disorders | 0.84 | 0.36 | 1 | Q13S4 |
| HINCOME_4 | 0.48 | 0.49 | 1 | Q27S1 |
| HINCOME_5 | 0.36 | 0.55 | 1 | Q28S1 |
| Avoid places where people gather | 0.07 | 0.78 | 1 | JOB_7 |
| Disinfect with hand washing, gargling and/or alcohol | 0.06 | 0.81 | 1 | Q15_2 |
| HINCOME_8 | 0.03 | 0.86 | 1 | HINCOME_3 |
| Avoid places with poor ventilation | 0.02 | 0.89 | 1 | HINCOME_8 |
| HINCOME_2 | 0.01 | 0.93 | 1 | JOB_3 |
| JOB_7 | 0.01 | 0.93 | 1 | Q6_binary |
| JOB_6 | 0.00 | 0.98 | 1 | JOB_6 |

| **S4-8. Mann-Whitney analysis for SAS positivity in 20's** | |  |  |  |
| --- | --- | --- | --- | --- |
| **Variables** | **p value** | **Mann-Whitney  U-stat** | **SAS+>SAS-** | **Question ID** |
| Excessive exercise before going to bed | 1.52E-50 | 134000.5 | TRUE | Q1S1 |
| Sleep in a room with the TV/light on | 1.34E-27 | 133446 | TRUE | Q1S4 |
| Sleep right after eating a meal | 2.21E-26 | 134224 | TRUE | Q1S3 |
| Main working situation | 3.71E-24 | 131772 | TRUE | Q24 |
| Drink alcohol before bedtime | 1.49E-23 | 132142.5 | TRUE | Q1S8 |
| BrinkmanIndex | 2.12E-22 | 50183.5 | FALSE | BrinkmanIndex |
| Oversleep | 3.43E-15 | 119167.5 | TRUE | Q1S6 |
| Too sleepy at work | 7.88E-15 | 44976.5 | FALSE | Q17_global_score |
| ESS_global_score | 1.24E-14 | 40994.5 | FALSE | ESS_global_score |
| Work performance | 1.95E-12 | 126583.5 | TRUE | Q16_global_score |
| PSQI_global_score | 1.32E-10 | 48693.5 | FALSE | PSQI_global_score |
| Take a bath within 90 minutes before bed | 3.26E-07 | 113442.5 | TRUE | Q1S7 |
| View the screen of your computer, smartphone or tablet before going to bed | 9.38E-07 | 67148 | FALSE | Q1S2 |
| Go to bed at different times | 3.19E-05 | 108990 | TRUE | Q1S5 |
| Working hours | 0.00061 | 66230 | FALSE | Q19_global_score |
| BMI | 0.0012 | 66874.5 | FALSE | BMI |
| Mood | 0.0083 | 70390 | FALSE | Q20_global_score |
| Melancholy | 0.0086 | 70782.5 | FALSE | Q21S1N |
| subjective_sleep_latency | 0.021 | 98929.5 | TRUE | subjective_sleep  _latency |
| AGE | 0.72 | 87751 | TRUE | AGE |
| subjective_sleep_duration | 0.81 | 87133 | FALSE | subjective_sleep  _duration |

**Table S5. Survey questionnaires**

|  |  |  |  |  |  |  |  |
| --- | --- | --- | --- | --- | --- | --- | --- |
| Survey Regarding Sleep   \|  \| \| --- \| |  |  |  |  |  |  |  |
|  |  |  |  |  |  |  |  |
|  |  |  |  |  |  |  |  |
|  |  | Meaning of Symbols | | |  |  |  |
|  |  | □ |  | Multiple Choice（Checkbox） |  |  |  |
|  |  | ○ |  | Single Selection（Radio Button） |  |  |  |
|  |  |  |  |  |  |  |  |
|  | **Q1** |  |  | With regards to sleep habits, please answer how often you… |  |  |  |
|  |  |  | 1. | Exercise excessively before going to bed | *Q1S1* |  |  |
|  |  |  | 2. | View the screen of your computer, smartphone or tablet before going to bed | *Q1S2* |  |  |
|  |  |  | 3. | Sleep right after eating a meal | *Q1S3* |  |  |
|  |  |  | 4. | Sleep in a room with the TV/light on | *Q1S4* |  |  |
|  |  |  | 5. | Go to bed at different times | *Q1S5* |  |  |
|  |  |  | 6. | Oversleep | *Q1S6* |  |  |
|  |  |  | 7. | Take a bath within 90 minutes before bed. Or no bath nor shower（Bathe in the morning） | *Q1S7* |  |  |
|  |  |  | 8. | Drink alcohol before bedtime | *Q1S8* |  |  |
|  |  |  |  |  |  |  |  |
|  |  | List of choices | | |  |  |  |
|  |  | ○ | 1. | Almost everyday |  |  |  |
|  |  | ○ | 2. | 3 or more times a week |  |  |  |
|  |  | ○ | 3. | Once or twice a week |  |  |  |
|  |  | ○ | 4. | None |  |  |  |
|  |  |  |  |  |  |  |  |
|  | **Q2** |  |  | Do you have a habit of smoking? |  |  |  |
|  |  | ○ | 1. | Yes |  |  |  |
|  |  | ○ | 2. | I used to smoke but quit |  |  |  |
|  |  | ○ | 3. | No |  |  |  |
|  |  |  |  |  |  |  |  |
|  | **Q3** |  |  | Please tell me how many cigarettes you smoke per day. |  |  |  |
|  |  |  | 1. | 【N】 cigarette(s) | *Q3S1N* |  |  |
|  |  |  |  |  |  |  |  |
|  | **Q4** |  |  | How many years have you been smoking since you started smoking habitually? |  |  |  |
|  |  |  | 1. | 【N】 year(s) | *Q4S1N* |  |  |
|  |  |  |  |  |  |  |  |
|  | **Q5** |  |  | How interested are you in quitting smoking? |  |  |  |
|  |  | ○ | 1. | Not interested |  |  |  |
|  |  | ○ | 2. | I'm thinking about it, but not right away |  |  |  |
|  |  | ○ | 3. | I'm thinking about quitting smoking immediately |  |  |  |

|  | **Q6** |  |  | During the past month, on average how much longer have you slept on weekends compared to weekdays? ※ Weekdays: Days when you have work. Weekends: Days when you do not have work. |  |  |  |
| --- | --- | --- | --- | --- | --- | --- | --- |
|  |  | ○ | 1. | Same |  |  |  |
|  |  | ○ | 2. | Less than 1 hour |  |  |  |
|  |  | ○ | 3. | 1-2 hours |  |  |  |
|  |  | ○ | 4. | 2-3 hours |  |  |  |
|  |  | ○ | 5. | 3-4 hours |  |  |  |
|  |  | ○ | 6. | More than 4 hours |  |  |  |
|  |  |  |  |  |  |  |  |
|  | **Q7** |  |  | Please answer the followings for the past month. |  |  |  |
|  |  |  | 1. | Time you went to bed | *Q7S1N/S2N* |  |  |
|  |  |  | 2. | Time needed to fall asleep | *Q7S3N/S4N* |  |  |
|  |  |  | 3. | Time you woke up | *Q7S5N/S6N* |  |  |
|  |  |  |  |  |  |  |  |
|  | **Q8** |  |  | During the past month, how often has your sleep been disturbed due to the following reasons: ※ If there are no "Other reason(s)", please respond with "None". |  |  |  |
|  |  |  | 1. | Could not fall asleep within 30 minutes of going to bed | *Q8S1* |  |  |
|  |  |  | 2. | Waking up in the middle of the night or early in the morning | *Q8S2* |  |  |
|  |  |  | 3. | Having to get up to use the bathroom | *Q8S3* |  |  |
|  |  |  | 4. | Having difficulty breathing | *Q8S4* |  |  |
|  |  |  | 5. | Coughing or loud snoring | *Q8S5* |  |  |
|  |  |  | 6. | Feeling too cold | *Q8S6* |  |  |
|  |  |  | 7. | Feeling too hot | *Q8S7* |  |  |
|  |  |  | 8. | Having bad dreams | *Q8S8* |  |  |
|  |  |  | 9. | Feeling pain | *Q8S9* |  |  |
|  |  |  | 10. | Other reason(s) | *Q8S10* |  |  |
|  |  |  |  |  |  |  |  |
|  |  | List of Choices | | |  |  |  |
|  |  | ○ | 1. | None |  |  |  |
|  |  | ○ | 2. | Less than once a week |  |  |  |
|  |  | ○ | 3. | 1-2 times a week |  |  |  |
|  |  | ○ | 4. | 3 or more times a week |  |  |  |
|  |  |  |  |  |  |  |  |
|  | **Q9** |  |  | How would you rate your sleep quality overall for the past month? |  |  |  |
|  |  | ○ | 1. | Very good |  |  |  |
|  |  | ○ | 2. | Fairly good |  |  |  |
|  |  | ○ | 3. | Fairly bad |  |  |  |
|  |  | ○ | 4. | Very bad |  |  |  |

|  | **Q10** | |  | During the past month, how often have you taken medicine to help you sleep? (Prescribed or OTC) |  |  |  |
| --- | --- | --- | --- | --- | --- | --- | --- |
|  |  | ○ | 1. | None |  |  |  |
|  |  | ○ | 2. | Less than once a week |  |  |  |
|  |  | ○ | 3. | 1-2 times a week |  |  |  |
|  |  | ○ | 4. | 3 or more times a week |  |  |  |
|  |  |  |  |  |  |  |  |
|  | **Q11** | |  | During the past month, how often have you had trouble staying awake while driving, eating meals, or engaging in social activity? |  |  |  |
|  |  | ○ | 1. | None |  |  |  |
|  |  | ○ | 2. | Less than once a week |  |  |  |
|  |  | ○ | 3. | 1-2 times a week |  |  |  |
|  |  | ○ | 4. | 3 or more times a week |  |  |  |
|  |  |  |  |  |  |  |  |
|  | **Q12** | |  | During the past month, how difficult has it been for you to sustain the motivation necessary to get things done? |  |  |  |
|  |  | ○ | 1. | No problem |  |  |  |
|  |  | ○ | 2. | Slight problems |  |  |  |
|  |  | ○ | 3. | Some problems |  |  |  |
|  |  | ○ | 4. | Considerable problems |  |  |  |
|  |  |  |  |  |  |  |  |
|  | **Q13** | |  | Please answer "Yes" or "No" to the following sleep questions: |  |  |  |
|  |  |  | 1. | Have you realized or been told by anyone that you snore while sleeping? | *Q13S1* |  |  |
|  |  |  | 2. | Have you realized or been told by anyone of stopped breathing while sleeping | *Q13S2* |  |  |
|  |  |  | 3. | Do you recently find yourself falling asleep before you realize? (for example, falling asleep during a meeting) | *Q13S3* |  |  |
|  |  |  | 4. | Do you still feel tired when you wake up? | *Q13S4* |  |  |
|  |  |  | 5. | Recently have you had to wake up at night to use the bathroom? | *Q13S5* |  |  |
|  |  |  | 6. | Has your weight increased by more than 10kg compared to when you were 20 years old? | *Q13S6* |  |  |
|  |  |  | 7. | Have you had rhinitis or bad teeth alignment from an early age? | *Q13S7* |  |  |
|  |  |  | 8. | In addition to snoring, do you or any of your relatives also have any of the following a) cardiovascular/cerebrovascular diseases, b) high blood pressure, c) hyperlipemia, or d) diabetes? | *Q13S8* |  |  |
|  |  |  |  |  |  |  |  |
|  |  | List of Choices | | |  |  |  |
|  |  | ○ | 1. | Yes |  |  |  |
|  |  | ○ | 2. | No |  |  |  |

|  | **Q14** | |  | Do you recognize yourself as "Short sleeper"? |  |  |  |
| --- | --- | --- | --- | --- | --- | --- | --- |
|  |  |  |  | Short sleeper: Those who have no physical disorder even with short sleep hours (less than 6 hours). |  |  |  |
|  |  | ○ | 1. | Yes |  |  |  |
|  |  | ○ | 2. | No |  |  |  |
|  |  |  |  |  |  |  |  |
|  | **Q15** | |  | Have you ever been diagnosed with and treated for a sleep disorder at a hospital or medical institution? If you answered yes, please select the applicable diagnosis. (Any number) |  |  |  |
|  |  | □ | 1. | Sleep apnea syndrome |  |  |  |
|  |  | □ | 2. | Other sleep disorder 【Free Answer】 |  |  |  |
|  |  | □ | 3. | No |  |  |  |
|  |  |  |  |  |  |  |  |
|  | **Q16** | |  | Please rate your "Work Performance" during the past month, with 100% being the best performance.  Similarly, please rate your condition for "Physical Health", "Mental Health", and "Brain Activity" over the past month in the same manner with the highest set at 100% |  |  |  |
|  |  |  | 1. | Performance of your work : 【N】％ | *Q16S1N* |  |  |
|  |  |  | 2. | Physical health：【N】％ | *Q16S2N* |  |  |
|  |  |  | 3. | Mental health : 【N】％ | *Q16S3N* |  |  |
|  |  |  | 4. | Brian activity : 【N】％ | *Q16S4N* |  |  |
|  |  |  |  |  |  |  |  |
|  | **Q17** | |  | How often during a single week have you been too sleepy to make progress at work? * Please input 0 if none |  |  |  |
|  |  |  | 1. | 【0-7】 day(s)/week | *Q17S1N* |  |  |
|  |  |  | 2. | 【0-24】 hour(s)/day | *Q17S2N* |  |  |

|  | **Q18** | |  | Please answer with your recent daily life in mind. How likely are you to doze off or fall asleep (a few seconds to a few minutes), in the following situations? Even if you haven’t been in these situations recently, try to imagine how they would have likely affected you. |  |  |  |
| --- | --- | --- | --- | --- | --- | --- | --- |
|  |  |  | 1. | Sitting and reading something | *Q18S1* |  |  |
|  |  |  | 2. | Sitting and watching TV | *Q18S2* |  |  |
|  |  |  | 3. | Sitting in a meeting, movie theater, theater, etc | *Q18S3* |  |  |
|  |  |  | 4. | As a passenger in a car continuously for an hour | *Q18S4* |  |  |
|  |  |  | 5. | Lying down in the afternoon to take a break or rest | *Q18S5* |  |  |
|  |  |  | 6. | Sitting and talking with someone | *Q18S6* |  |  |
|  |  |  | 7. | Sitting quietly after having lunch (without alcohol) | *Q18S7* |  |  |
|  |  |  | 8. | Sitting and writing letters or documents | *Q18S8* |  |  |
|  |  |  |  |  |  |  |  |
|  |  | List of Choices | | |  |  |  |
|  |  | ○ | 1. | High chance |  |  |  |
|  |  | ○ | 2. | Moderate chance |  |  |  |
|  |  | ○ | 3. | Slight chance |  |  |  |
|  |  | ○ | 4. | Never |  |  |  |
|  |  |  |  |  |  |  |  |
|  | **Q19** | |  | During the past month, please respond with how many hours have committed for the following: * Please deduct your break time from the following hours. * Please make sure that the total value of working hours and overtime hours is less than 24 hours in a day |  |  |  |
|  |  |  | 1. | Work hours | *Q19S1N* |  |  |
|  |  |  | 2. | Overtime work hours per day | *Q19S2N* |  |  |
|  |  |  |  |  |  |  |  |
|  |  |  |  |  |  |  |  |
|  | **Q20** | |  | Please tell us your frequency of experiencing the following moods and conditions during the past month |  |  |  |
|  |  |  | 1. | Feeling motivated | *Q20S1* |  |  |
|  |  |  | 2. | Feeling energetic | *Q20S2* |  |  |
|  |  |  | 3. | Feeling lively | *Q20S3* |  |  |
|  |  |  | 4. | Feeling angry | *Q20S4* |  |  |
|  |  |  | 5. | Feeling annoyed | *Q20S5* |  |  |
|  |  |  | 6. | Feeling irritated | *Q20S6* |  |  |
|  |  |  | 7. | Feeling tired | *Q20S7* |  |  |
|  |  |  | 8. | Feeling exhausted | *Q20S8* |  |  |
|  |  |  | 9. | Feeling dull | *Q20S9* |  |  |
|  |  |  | 10. | Feeling under pressure | *Q20S10* |  |  |
|  |  |  | 11. | Feeling anxious | *Q20S11* |  |  |
|  |  |  | 12. | Feeling restless | *Q20S12* |  |  |
|  |  |  | 13. | Feeling depressed | *Q20S13* |  |  |
|  |  |  | 14. | Not feeling like doing anything | *Q20S14* |  |  |
|  |  |  | 15. | Unable to focus | *Q20S15* |  |  |
|  |  |  | 16. | Not feeling well | *Q20S16* |  |  |
|  |  |  | 17. | Unable to concentrate on work | *Q20S17* |  |  |
|  |  |  | 18. | Feeling sad | *Q20S18* |  |  |
|  |  |  | 19. | Feeling dizzy | *Q20S19* |  |  |
|  |  |  | 20. | Having joint pains | *Q20S20* |  |  |
|  |  |  | 21. | Having a headache | *Q20S21* |  |  |
|  |  |  | 22. | Having neck/shoulder pains | *Q20S22* |  |  |
|  |  |  | 23. | Having back pains | *Q20S23* |  |  |
|  |  |  | 24. | Having eye strain | *Q20S24* |  |  |
|  |  |  | 25. | Having palpitations | *Q20S25* |  |  |
|  |  |  | 26. | Having gastrointestinal problems | *Q20S26* |  |  |
|  |  |  | 27. | Loss of appetite | *Q20S27* |  |  |
|  |  |  | 28. | Having constipation or diarrhea | *Q20S28* |  |  |
|  |  |  | 29. | Trouble sleeping | *Q20S29* |  |  |
|  |  |  |  |  |  |  |  |
|  |  | List of Choices | | |  |  |  |
|  |  | ○ | 1. | Rarely |  |  |  |
|  |  | ○ | 2. | Sometimes |  |  |  |
|  |  | ○ | 3. | Often |  |  |  |
|  |  | ○ | 4. | Almost always |  |  |  |

|  | **Q21** | |  | How many days out of the past week have you felt melancholy about going to work? * Please answer 0, if none. |  |  |  |
| --- | --- | --- | --- | --- | --- | --- | --- |
|  |  |  | 1. | 【N】 days per week | *Q21S1N* |  |  |
|  |  |  |  |  |  |  |  |
|  | **Q22** | |  | Please answer your occupation. (Select one) * If you have a more than one occupation, please select your main occupation. |  |  |  |
|  |  | ○ | 1. | CEO、COO、CFO、CIO、CTO、Executive manager、General manager、Branch manager、or any other senior position |  |  |  |
|  |  | ○ | 2. | Sales (business) |  |  |  |
|  |  | ○ | 3. | Counter sales |  |  |  |
|  |  | ○ | 4. | Customer services |  |  |  |
|  |  | ○ | 5. | Other |  |  |  |
|  |  | ○ | 6. | General office work, Assistant, Receptionist, Secretary, other related office work |  |  |  |
|  |  | ○ | 7. | Finance / Accounting / Auditing |  |  |  |
|  |  | ○ | 8. | General affairs, HR, Legal, Intellectual property, Public relations, IR |  |  |  |
|  |  | ○ | 9. | Other |  |  |  |
|  |  | ○ | 10. | Planning (Product planning, Sales planning and Corporate planning, etc.) |  |  |  |
|  |  | ○ | 11. | Marketing (Advertising/Promotion, PR, Brand manager/Product manager, Data analysis/Market research, Web/Digital marketing, etc.) |  |  |  |
|  |  | ○ | 12. | Service/Sales/Dining |  |  |  |
|  |  | ○ | 13. | Web/Internet/Games |  |  |  |
|  |  | ○ | 14. | Designer/Creator/Illustrator |  |  |  |
|  |  | ○ | 15. | Editing/Writer/Producer |  |  |  |
|  |  | ○ | 16. | Other |  |  |  |
|  |  | ○ | 17. | Programmer/Web engineer |  |  |  |
|  |  | ○ | 18. | In-house system engineer |  |  |  |
|  |  | ○ | 19. | Product development/ASP |  |  |  |
|  |  | ○ | 20. | Other |  |  |  |
|  |  | ○ | 21. | Engineer (design/manufacturing/quality control) |  |  |  |
|  |  | ○ | 22. | Technical expert (Materials/Chemical/Food/Pharmaceutical) |  |  |  |
|  |  | ○ | 23. | Technical expert (Construction/Civil engineering) |  |  |  |
|  |  | ○ | 24. | Skilled workers/Facilities/Traffic /Transportation |  |  |  |
|  |  | ○ | 25. | Teaching professions/Government worker/Agricultural, Forestry and fisheries |  |  |  |
|  |  | ○ | 26. | Consultant |  |  |  |
|  |  | ○ | 27. | Medical |  |  |  |
|  |  | ○ | 28. | Other |  |  |  |
|  |  |  |  |  |  |  |  |
|  | **Q23** | |  | Please answer the type of industry you work for (Only one thing) * If you have more than one occupation, please select your main industry. |  |  |  |
|  |  | ○ | 1. | Manufacturing |  |  |  |
|  |  | ○ | 2. | Trading |  |  |  |
|  |  | ○ | 3. | Distribution/Retail/Wholesale |  |  |  |
|  |  | ○ | 4. | Information/Communications |  |  |  |
|  |  | ○ | 5. | Media/Advertising |  |  |  |
|  |  | ○ | 6. | Finance/Insurance |  |  |  |
|  |  | ○ | 7. | Real estate/Construction |  |  |  |
|  |  | ○ | 8. | Logistics/Warehousing/Transportation |  |  |  |
|  |  | ○ | 9. | Consulting/Professional services |  |  |  |
|  |  | ○ | 10. | Medical/Pharmaceutical |  |  |  |
|  |  | ○ | 11. | Education/Human resources |  |  |  |
|  |  | ○ | 12. | Environment/Resources/Energy |  |  |  |
|  |  | ○ | 13. | Public corporations/government agencies |  |  |  |
|  |  | ○ | 14. | Agriculture/Fisheries |  |  |  |
|  |  | ○ | 15. | Other |  |  |  |

|  | **Q24** | |  | Please tell us about your current main working situation. Do you work from home? If so, please indicate how often you work from home. |  |  |  |
| --- | --- | --- | --- | --- | --- | --- | --- |
|  |  | ○ | 1. | Almost everyday |  |  |  |
|  |  | ○ | 2. | 3 or more times a week |  |  |  |
|  |  | ○ | 3. | Once or twice a week |  |  |  |
|  |  | ○ | 4. | Less than once a week |  |  |  |
|  |  | ○ | 5. | Never worked from home |  |  |  |
|  |  |  |  |  |  |  |  |
|  | **Q25** | |  | Do you currently have children? (Select all that apply) |  |  |  |
|  |  | □ | 1. | Preschooler (0-6 years old) |  |  |  |
|  |  | □ | 2. | Elementary school child |  |  |  |
|  |  | □ | 3. | Junior high school or high school child |  |  |  |
|  |  | □ | 4. | Professional student or college/graduate student child |  |  |  |
|  |  | □ | 5. | Child in work force |  |  |  |
|  |  | □ | 6. | Other |  |  |  |
|  |  | □ | 7. | No children |  |  |  |
|  |  |  |  |  |  |  |  |
|  | **Q26** | |  | Please answer your height and weight |  |  |  |
|  |  |  | 1. | Height 【N】 | *Q26S1N* |  |  |
|  |  |  | 2. | Weight 【N】 | *Q26S2N* |  |  |
|  |  |  |  |  |  |  |  |
|  | **Q27** | |  | Please answer whether the following symptoms that apply to you. |  |  |  |
|  |  |  | 1. | From 2020 to the present, I have felt some kind of illness | *Q27S1* |  |  |
|  |  |  | 2. | From 2020 to the present, I experienced cold symptoms (sore throat, cough, sputum, fever, etc.) | *Q27S2* |  |  |
|  |  |  | 3. | From 2020 to the present, I received the influenza vaccine | *Q27S3* |  |  |
|  |  |  | 4. | From 2020 to the present, I got the flu | *Q27S4* |  |  |
|  |  |  | 5. | Due to suspicion of COVID-19, I went to the hospital | *Q27S5* |  |  |
|  |  |  | 6. | Due to suspicion of COVID-19、I was hospitalized | *Q27S6* |  |  |
|  |  |  | 7. | Due to suspicion of COVID-19、I was admitted to a hotel for treatment and recuperation | *Q27S7* |  |  |
|  |  |  | 8. | Due to suspicion of COVID-19、I underwent home quarantine | *Q27S8* |  |  |
|  |  |  | 9. | Voluntarily isolation due to close contact with COVID-19 | *Q27S9* |  |  |
|  |  |  | 10. | Family members, relatives, friends, or colleagues at work, have been diagnosed with COVID-19 | *Q27S10* |  |  |
|  |  |  |  |  |  |  |  |
|  |  | List of Choices | | |  |  |  |
|  |  | ○ | 1. | Yes |  |  |  |
|  |  | ○ | 2. | No |  |  |  |
|  |  | ○ | 3. | Do not want to answer |  |  |  |
|  |  |  |  |  |  |  |  |
|  | **Q28** | |  | Please tell us what methods you have applied for prevention of infection from COVID-19. [Yes or No response required for all items aside from "Others"] |  |  |  |
|  |  |  | 1. | I try to avoid places with poor ventilation | *Q28S1* |  |  |
|  |  |  | 2. | I try to avoid places where people gather | *Q28S2* |  |  |
|  |  |  | 3. | I try not to have conversations with other people in close proximity | *Q28S3* |  |  |
|  |  |  | 4. | I actively disinfect with hand washing, gargling and/or alcohol | *Q28S4* |  |  |
|  |  |  | 5. | I always wear a mask when going out | *Q28S5* |  |  |
|  |  |  | 6. | I avoid unnecessary and non-essential outings | *Q28S6* |  |  |
|  |  |  | 7. | I have been telecommuting | *Q28S7* |  |  |
|  |  |  | 8. | I purchased antibacterial goods that I have never bought before | *Q28S8* |  |  |
|  |  |  | 9. | Other | *Q28S9* |  |  |
|  |  |  |  |  |  |  |  |
|  |  | List of Choices | | |  |  |  |
|  |  | ○ | 1. | Yes |  |  |  |
|  |  | ○ | 2. | No |  |  |  |
